# Allogeneic MHC-mismatched microglia-like cell replacement as a therapeutic approach for multiple sclerosis

**DOI:** 10.1186/s12974-025-03672-4

**Published:** 2026-01-08

**Authors:** Irene Benito-Cuesta, Jin-Hong Min, Yuxi Guo, Giulia Adriana Virgilio, Valerie Suerth, Stefan Bencina, Paula Trigo-Alonso, Keying Zhu, Shin-Yu Kung, Majid Pahlevan Kakhki, Heela Sarlus, Robert A. Harris

**Affiliations:** 1 Department of Clinical Neuroscience, Karolinska Institutet, Center for Molecular Medicine, Karolinska University Hospital Solna, Stockholm, Sweden; 2 Goethe University Frankfurt, Frankfurt am Main, Germany; 3 Instituto Teófilo Hernando for Drug Discovery, Department of Pharmacology, School of Medicine, Universidad Autónoma de Madrid, Madrid, Spain

**Keywords:** Microglia replacement, Adoptive transfer, Allogeneic, MHC-mismatch, EAE, Multiple sclerosis, Microglia-like cells, BALB/c, C3H

## Abstract

**Background:**

Dysfunctional microglia contribute to the pathology of numerous neurological diseases. Depletion of harmful microglia and repopulation with healthy progenitors represents a new therapeutic option for neurodegenerative diseases with an urgent need for effective treatments. However, repopulation with patient-derived progenitors could result in similar dysfunction over time. We therefore propose obtaining microglia-like cells (MLCs) derived from healthy donors for allogeneic transplantation. We hypothesize that the immunosuppressive phenotype of MLCs, combined with the brain´s high immune tolerance, would enable effective engraftment. Additionally, the allogeneic origin of MLCs may increase immune tolerance, with additional therapeutic outcomes, particularly in multiple sclerosis (MS).

**Methods:**

MLCs were generated from MHC-mismatched mouse strains in vitro and exposed to IL-10/IL-4/TGF-β or LPS/IFN-γ to induce specific immunophenotypes. Phagocytosis and T cell proliferation assays assessed MLC responses to pathogenic insults that could arise in autoimmune contexts. Microglial depletion was achieved using Cx3cr1^CreER/−^R26^DTA/−^ mice or PLX3397 treatment. A protocol administering MLCs directly into the brain via the intracisterna magna was optimized to facilitate repopulation of the microglial niche. Repopulation with MHC-mismatched IL-10/IL-4/TGF-β-polarized MLCs was tested in the experimental autoimmune encephalomyelitis (EAE) mouse model of MS, with immune profiling of cellular populations conducted using flow cytometry.

**Results:**

BALB/c- and C3H-derived MLCs developed more pronounced anti-inflammatory profiles than did C57BL/6 MLCs, and promoted tolerogenic phenotypes when encountering MHC-mismatched C57BL/6 T cells. In vivo experiments demonstrated a partial repopulation of an emptied microglia-niche by pre-differentiated MLCs administered intracisternally (i.c) into the CNS. The adoptive transfer of MHC-mismatched MLCs led to enhanced immune tolerance mechanisms and the amelioration of disease progression in the EAE mouse model.

**Conclusions:**

Our findings support the therapeutic potential of anti-inflammatory MHC-mismatched MLCs in promoting immune tolerance within autoimmune neuropathologies. Specifically, disease progression was attenuated and tolerogenic mechanisms were activated in the MS mouse model. While a polarization protocol towards an anti-inflammatory phenotype confers MLCs with beneficial features in a pro-inflammatory disease context, the MHC-mismatch interaction within the host´s CNS promotes additional tolerogenic processes.

**Supplementary Information:**

The online version contains supplementary material available at 10.1186/s12974-025-03672-4.

## Background

Microglia comprise approximately 10% of non-neuronal cells in the central nervous system (CNS). As the brain’s resident immune cells, microglia are responsible for several critical functions including surveillance, phagocytosis of harmful debris, mediating inflammation through the secretion of cytokines and chemokines, and acting as antigen-presenting cells (APCs). Additionally, microglia play a key role in maintaining homeostasis by participating in processes such as vasculogenesis, blood-brain barrier (BBB) permeability, remyelination, neurogenesis and synapse pruning [1]. However, many neurological disorders are associated with dysfunctional microglia, resulting in significant pathogenic effects [2–4]. Due to their versatility and critical role in these conditions, microglia have become an interesting therapeutic target for treating neurodegenerative diseases linked to microglial dysfunction.

While approaches to target microglia are already being tested in clinical trials, microglia replacement therapies represent a new therapeutic option for neurodegenerative diseases such as Amyotrophic Lateral Sclerosis (ALS) [5] or Multiple Sclerosis (MS) [6]. The underlying therapeutic principle involves resetting the microglial niche by depleting dysfunctional microglia, thereby allowing newly repopulating microglia to populate, resolve ongoing inflammation and promoting functional recovery. Depletion strategies have shown promising results in several experimental neurological disorders [7]. However, repopulation from endogenous progenitors may only provide temporary benefits, as the new microglial cells could eventually develop similar dysfunction with time. Although encouraging, this strategy remains insufficient and misses the opportunity for a truly long-lasting therapeutic effect.

The adoptive transfer of microglial cells offers greater control over microglia replacement therapies and moves us towards personalized treatments. However, the unique origin of microglia in the embryonic yolk sac and their eventual localization in the nervous system [8, 9] complicates their isolation from human donors. The administration of peripheral myeloid precursors presents a viable alternative for enhancing the repopulation of the microglial niche [7]. Engrafting into the CNS leads to myeloid precursors acquiring a microglia-like cell (MLC) phenotype, which shares about 90% of the transcriptome with host microglia [10]. However, MLCs derived from a patient’s own monocytes could carry mutations linked to the disease progression such as in ALS, making autologous transplantation potentially counterproductive. In this case allogeneic transplantation of MLCs derived from healthy donors could be essential to achieve therapeutic effects.

Multiple Sclerosis is the most common chronic inflammatory, demyelinating and neurodegenerative disease of the CNS in young adults [6]. Influenced by both genetic and environmental factors, MS is primarily characterized by the presence of multifocal acute lesions with reactive gliosis, BBB breakdown and infiltration of autoreactive lymphocytes. Clusters of ramified microglia precede MS lesion formation (preactive lesions) in normal appearing white matter (NAWM). These later develop into active lesions with amoeboid microglia (known as *microglia inflamed in MS*, MIMS), which remain at the chronic active lesion edge as they expand [3, 4]. While the *MIMS-Foamy* phenotype has been associated with repair functions, the *MIMS-iron* phenotype contributes to the direct propagation of inflammatory damage by acting as APCs for T cells, releasing pro-inflammatory cytokines and reactive oxygen species (ROS), and inhibiting oligodendrocyte precursor cell (OPC) differentiation [4, 11].

Herein we propose the allogenic transplantation of anti-inflammatory MLCs into a microglia-depleted CNS as a therapeutic strategy for MS. A previously established protocol permits the differentiation of patient peripheral monocytes into MLCs [12], and a stable anti-inflammatory profile can be induced [13] for their subsequent i.c adoptive transfer directly into the CNS. We hypothesize that the induced immunosuppressive phenotype, coupled with the higher immune tolerance of the CNS [14], would prevent the immune rejection of transplanted MLCs. Moreover, the allogeneic origin of MLCs may further promote immune tolerance, offering additional therapeutic effects in autoimmune diseases such as MS [15].

To test our hypothesis, we selected MHC-mismatched mouse strains to generate MLCs, which developed different immunomodulatory profiles when stimulated in vitro. In vivo experiments demonstrated a partial repopulation of an emptied microglia-niche by pre-differentiated MLCs administered i.c into the CNS. The adoptive transfer of MHC-mismatched MLCs led to the enhancement of immune tolerance mechanisms and the therapeutic amelioration of EAE disease progression.

## Methods

### Experimental animals

All mice were bred and maintained at Karolinska Institutet under specific pathogen-free and climate-controlled conditions with regulated 12 h light/dark cycles and *ad libitum* access to food and water, in accordance with national animal care guidelines. All animal experiments were approved by the appropriate ethical review board (Stockholms djurförsöksetiska nämnd). Cx3cr1^CreER − EYFP^R26^DTA^ were originally obtained from The Jackson Laboratory; C57BL/6NTac mice from Taconic; BALB/cAnNCtrl (BALB/c) and C3H/HeNcrl (C3H) from Charles River. CD45.1 mice were obtained from F. Wermeling and E. Villablanca (Karolinska Institutet). B6.2D2 mice were obtained from AO Guerreiro-Cacais. Homozygous Cx3cr1^CreER/CreER^ (CD45.2/CD45.2) and R26^DTA/DTA^ (CD45.2/CD45.2) were crossed to generate Cx3cr1^CreER/−^R26^DTA/−^ (CD45.2/CD45.2). Cx3cr1^CreER/−^R26^DTA/−^ (CD45.2/CD45.2) were crossed with R26^DTA/DTA^ (CD45.2/CD45.2) to generate Cx3cr1^CreER/−^R26^DTA/DTA^ (CD45.2/CD45.2). Cx3cr1^CreER/−^R26^DTA/DTA^ (CD45.2/CD45.2) were backcrossed to generate homozygous Cx3cr1^CreER/CreER^R26^DTA/DTA^ (CD45.2/CD45.2). Cx3cr1^CreER/CreER^R26^DTA/DTA^ (CD45.2/CD45.2) were crossed with CD45.1/CD45.1 mice to generate Cx3cr1^CreER/−^R26^DTA/−^ CD45.2/CD45.1 mice. Experiments were generally initiated when mice were 4 months-old.

### MLC differentiation and treatments

Femurs obtained from C57BL/6, C57BL/6^GFP+^, BALB/c or C3H mice were flushed with PBS and bone marrow (BM) resuspended and filtered through a 40 μm nylon strainer. The resulting single-cell suspensions were centrifuged at 350 g for 5 min at 4 °C and the pellets were resuspended in complete medium (Dulbecco’s Modified Eagle (DMEM)/F12 medium (Gibco, 31331093), 2mM l-Glutamine (Sigma-Aldrich, G7513), 1mM sodium pyruvate (Sigma, S8636), and 20µM 2-Mercaptoethanol (Gibco, 31350-010)), 50U/mL penicillin and 50 µg/mL streptomycin (Sigma-Aldrich, P4458)). Complete media was supplemented with 10% heat-inactivated fetal bovine serum (FBS; Sigma-Aldrich, F7524), 50ng/ml IL-34 (R&D systems, 5195-ML-010) and 10ng/ml granulocyte-macrophage colony stimulating factor (GM-CSF) (R&D systems, 415-ML-010) to promote MLC differentiation. Cells were cultured at 37 °C and 5% CO_2_ in a T175-cell culture flask (Sarstedt, 83.3912.002) containing 20mL of media for each 2 femurs. Media was changed every 3–4 days.

Differentiated MLCs were harvested after 6–7 days using trypsin/EDTA solution (Gibco, 25300096), and plated at a density of approximately 1.5 × 10^5^ cells/cm^2^ for subsequent in vitro experiments. MLCs were cultured in complete medium + 10% FBS and supplemented for 24 h either with (i) 50ng/mL LPS and 20ng/mL IFN-γ (R&D systems, 485-ML-100) to promote a proinflammatory phenotype (M1), or (ii) 20ng/ml IL-10 (R&D systems, 417-ML-005), 20ng/ml IL-4 (R&D systems, 404-ML-010) and 20ng/ml TGF-b 1 (R&D systems, 7666-MB-005) to promote an anti-inflammatory phenotype (M2). Unstimulated MLCs (M0) were maintained in complete medium + 10% FBS for the same periods.

### Reverse transcription polymerase chain reaction (RT-qPCR)

RNA from cultured cells was obtained using the RNeasy mini kit (Qiagen, 74106) with on-column DNase I digestion (Qiagen, 79254) according to the manufacturer’s instructions. IScript kit (BioRad Laboratories, 1708891) was used for reverse transcription to cDNA. SYBR green (BioRad Laboratories, 1708886) was used for mRNA amplifications in BioRad CFX384 Touch Real-Time PCR Detection System. All expression levels are reported relative to *Hprt* and *Gapdh*. Primer sequences are listed in Additional file 1: Table S1.

### Phagocytosis

MLCs were incubated with *E. coli* particles (20 µg/ml, Invitrogen, P35361) for 20 min in complete medium. Cells were detached thereafter and labelled for flow cytometry analysis (BD LSR Fortessa) as described.

Alternatively, MLCs were plated in Incucyte^®^ Imagelock 96-well Plates (Sartorius, BA-04856) and, after applying the 24 h polarization protocol, incubated in the presence of Zymosan particles (Invitrogen, P35364) at a concentration of 0.1 mg/ml in complete medium + 0.1% FBS for 24 h in the live cell imaging system Incucyte S3. Analysis was performed using Incucyte S3 software.

### T cell proliferation

Spleens from C57BL/6 mice were mechanically disrupted through a 40 μm nylon strainer. Incubation with ACK buffer (Gibco) for 5 min was used to lyse erythrocytes. T lymphocytes were isolated using the Pan T Cell Isolation Kit II (Mitenyi Biotec, 130-095-130) and LS Columns (Miltenyi Biotec 130-042-401) in a MidiMACS Separator (Miltenyi Biotec).

T lymphocytes were labelled with CFSE (Biolegend, 423801) following the manufacturer’s instructions. MLCs and T cells were cocultured with 1 µg/ml anti-CD3 (BD Biosciences 555273) and 2 µg/ml anti-CD28 (BD Biosciences, 553294) in RPMI supplemented with 10% FBS, 2mM l-Glutamine, 1mM sodium pyruvate, 20µM 2-Mercaptoethanol, 50U/mL penicillin and 50 µg/mL streptomycin. 72 h later, T cells were collected for staining and analysis by flow cytometry.

Alternatively, 20 µg/ml MOG(35–55) peptide (Anaspec, AS-60130-10) was used as a stimulus for T cells collected from B6.2D2 mice.

### *In vivo* microglia depletion

Tamoxifen (TAM; Sigma, T5648) was dissolved in corn oil at 75 °C for 1 h at a concentration of 20 mg/ml. Cx3cr1^CreER − EYFP/−^ R26^DTA/−^ mice (or C57BL/6 as negative control of microglia depletion) were administered intraperitoneally (i.p) with 4 mg tamoxifen per mouse on three consecutive days. In EAE mice, tamoxifen was administered at the onset of disease from day 9–11.

Alternatively, microglia were depleted by feeding mice with chow containing PLX3397 (Pexidartinib, 290 mg/kg) for 21 consecutive days. Chow diet containing PLX3397 (MedChemTronica) was formulated by SAFE^®^ Complete Care Competence.

### Intracisternal injection of MLCs

MLCs were detached using trypsin/EDTA solution, washed with PBS and centrifuged at 350 g for 5 min at 4 °C. MLCs were resuspended in PBS to obtain 50 × 10^6^ cells/mL, unless otherwise specified. Intracisternal injection was performed as previously described [16]. Briefly, the tip of a 27G dental needle (Terumo, DN-2721) was bent (around 3.5 mm) at an angle of 40°. The short end of the needle was connected with a Hamilton syringe via a polyethylene tube. Mice were anesthetized with isoflurane (Baxter, 1001936040) and leaned on a box, bending the head slightly forward. The bent end of the needle was inserted into the cleft between the occiput and the atlas vertebra, and 10µL of solution was slowly injected during approximately 10s. Mice were removed from anesthesia after extracting the needle and monitored to ensure their rapid recovery. According to the veterinary guidelines, no analgesia was needed during or after the procedure.

For tracking experiments, MLCs pre-labelled with CellTrace™ Violet (Thermo Fisher Scientific) according to the manufacturer’s instructions were injected i.c as described above.

### Induction of experimental autoimmune encephalomyelitis (EAE)

Mouse recombinant myelin oligodendrocyte glycoprotein (rMOG, amino acids 1–125 from the N terminus) was expressed in *E. coli* and purified to homogeneity by chelate chromatography. Purified rMOG dissolved in 6 M urea was then dialyzed against sodium acetate buffer (10mM, pH3.0) to obtain a soluble preparation. For EAE induction, mice were anesthetized with isoflurane and immunized subcutaneously in the dorsal tail base with 100 µl solution containing 40 µg rMOG emulsified in complete Freund’s adjuvant (Chondrex, 7027) (200 µg Mycobacterium tuberculosis per mouse). rMOG emulsion was prepared using the POWER-Kit according to the manufacturer’s instructions (BTP emulsion, Malmö, Sweden, https://btbemulsions.com). Mice received i.p injections of 200ng pertussis toxin (Sigma, P7208) on the day of immunization and 48 h later. Body weight and paralysis were monitored from day 8 post-immunization, and wet food was changed daily. The paralytic symptoms of EAE were scored according to the following criteria: 0 = no clinical signs of EAE; 0.5 = tail weakness, 1 = tail paralysis, 1.5 = hindlimb hemiparesis or weakness, 2 = hindlimb paraparesis, 2.5 = hindlimb hemiparalysis, 3 = hindlimb paralysis, 3.5 = hindlimb paralysis with forelimb paraparesis, 4 = tetraplegia or moribund, 5 = death. Mice were euthanized if they exhibited > 25% weight loss or reached tetraplegia. Mice showing clinical symptoms (score > 0.5) were included in the analysis of EAE paralysis progression.

### Isolation of cells from brain/spinal cords

Mice were anesthetized with pentobarbital or isoflurane to perform cardiac perfusion with 20 mL PBS. Hemibrains or spinal cords were dissected and mechanically dissociated. Homogenates were incubated in 5mL papain (Worthington, LS003126; 1:100 diluted in L15 medium) supplemented with DNase I (Roche, 10104159001, 0.2 mg/mL) at 37℃ for 20 min, with mechanical dissociation every 10 min. Homogenates were passed through 40 μm cell strainers and 20mL cold HBSS was added to terminate the enzymatic reaction and wash. After centrifugation at 350 g for 5 min at 4℃, the cell pellets were resuspended in 15mL 37% isotonic Percoll (Sigma-Aldrich, GE17-0891-01) in HBSS and centrifuged at 800 g (acceleration 4, deceleration 0) for 10 min. Cell pellets were washed with PBS and pelleted at 350 g for 5 min at 4 °C for downstream analysis.

For intracellular cytokine accumulation, isolated cells were incubated 3–5 h at 37 C in 50ng/ml Phorbol-12-myristate-13-acetate (PMA, Sigma, 524400), 1 µg/ml ionomycin (Sigma, I0634) and 1 µg/ml GolgiPlug™ (BD, 555029) in 200 µl/well complete RPMI medium + 10% FBS.

### Flow cytometry analysis

Single cell suspensions from tissues or cultured cells were stained with Live/Dead Fixable Near-IR (Invitrogen, L34976) or Yellow Dead Cell Stain Kit (Invitrogen, L34959) 1:2000 in the presence of Fc Block (BD Biosciences, 553142, 1:200) for 15 min. After washing, cells were incubated with antibodies for 30 min. When performing intracellular staining, cells were treated with fixation/permeabilization buffer (eBioscience, 00-5523-00) for 1–16 h followed by incubation with antibodies for the intracellular compartment for 45 min. Otherwise, cells were fixed in paraformaldehyde (PFA) 2% for 10 min. Cells were acquired using Aurora (Cytek) and analyzed using Kaluza 2.1 software (Beckman Coulter). Cellular populations were gated according to Fluorescence Minus One controls (FMOs) as well as negative and positive staining controls. An antibody list is included in Additional file 1: Table S2.

### Immunofluorescent staining

Perfused hemibrains and spinal cords were immersion-fixed in 4% PFA for 24 h, then immersed in 15% sucrose solution for 24 h and in 30% sucrose solution for at least 24 h more. Tissues were then embedded in OCT Cryomount (Histolab, 45830), frozen in dry ice and sectioned at 14 μm. Sections were washed in Tris-buffered saline (TBS, 50mM Tris-HCl in 150mM NaCl, pH7.5), blocked and permeabilised with 1% Triton X-100 1% BSA TBS solution, stained with anti-Iba1 (Wako chemicals, 019-19741) antibody overnight, washed and incubated with goat anti-rabbit Alexa Fluor 594 (Invitrogen, A11037) for 1 h. FluoroMyelin Red staining (1:500) of spinal cords was performed during the secondary antibody incubation step, followed by 20 min Hoechst staining (1:10000 in PBS). Images were acquired using a Zeiss LSM800 confocal microscope and analyzed using Image J software.

Cultured MLCs in black wall/glass bottom plates were fixed in 4% PFA for 20 min. A similar staining protocol was used, with the additional incubation in Hoechst (Thermo Fisher Scientific, 62249, 1:5000) for 15 min for nuclei visualization.

### Statistical data analysis

GraphPad Prism 9.5.1 and R version 4.4.1 softwares were used to perform statistical analysis and to generate graphs. Comparison between two groups was performed with Student’s two-tailed unpaired t test. For multiple comparisons one-way ANOVA with subsequent Tukey’s multiple comparison test was performed. *P* < 0.05 was considered statistically significant.

### Cell sorting

The Neural Tissue Dissociation kit T (Miltneyi Biotec ,130-093-231) was used to generate brain homogenates according to manufacturer’s protocol. Subsequently, the homogenates were treated with Percoll for myelin removal as described above. The cell suspensions from 3 mice were pooled and then stained with anti-mouse CD45-BUV496 (clone 30-F1, 1:200), CD11b-Percp-Cy5,5 (clone M1/70, 101228, Biolegend), Ly6C-AF700 (clone HK1.4, 128024, Biolegend), Ly6G-AF647 (clone 1A8, 127609, Biolegend), F4/80-BV421 (clone BM8, 123137, Biolegend), and Live/Dead Fixable Near-IR Cell Stain Kit (Invitrogen, L34976, 1.500). Microglia in these mice express YFP under CX3CR1-promoter [10]. MLCs were sorted as CD45^+^CD11b^+^F4/80^high^Ly6G^−^Ly6C^−^GFP^+^ cells and Cytek Aurora cell sorter. Endogenous microglia sorted as CD45^+^CD11b^+^F4/80^high^Ly6G^−^Ly6C^−^GFP^−^YFP^+^ were used as controls. Subsequently, the sorted cells were washed with PBS, centrifuged at 350 g for 5 min at 4 °C, and resuspended in lysis buffer and stored at -80 °C.

### Bulk RNAseq and data analysis

Total RNA was extracted from sorted MLCs and microglia respectively using the RNeasy Micro Kit (QIAGEN, Cat. No. 74004) according to the manufacturer’s instructions optimized for low-input material. RNA integrity and concentration were assessed using an Agilent Bioanalyzer (Agilent Technologies), and all samples exhibited RNA integrity numbers (RIN) greater than 6. RNA-sequencing libraries were prepared using the Xpress-seq bulk RNA-seq workflow (Xpress Genomics AB, Solna, Sweden). Oligo(dT)-primed reverse transcription was performed to selectively capture polyadenylated mRNA transcripts, and library construction followed the manufacturer’s low-input RNA protocol. Libraries were sequenced on an Illumina platform to a depth of 25–50 million paired-end reads per sample. Sequencing was performed using standard Illumina chemistry, and resulting reads were demultiplexed and quality-trimmed. Quality-filtered reads were aligned to the mouse reference genome (mm38) using STAR [17] with default parameters, and unmapped reads were retained in the resulting BAM files. Read quality was assessed using FastQC, and gene-level quantification was performed with featureCounts from the Rsubread package, applying GENCODE gene annotations.

Genes with more than 5 counts in over 3 samples were retained, resulting in 16,363 genes for downstream analysis. R package DESeq2 (version 1.48.2) was used to identify differentiated gene expression. R packages EnhancedVolcano (version 1.26.0) and ggplot2 were used to make volcano plot. Gprofiler2 (version 0.2.3) and enrichplot (version 1.28.4) were used to calculation enriched gene ontology terms and their visualization. edgeR (version 4.6.3) and limma (version 3.64.3) were sued to generate normalized, batch effect removed counts then ComplexHeatmap (version 2.24.1) was further used for making heatmap.

## Results

### MLCs injected into cisterna magna partially repopulate the brain parenchyma following microglia depletion

We proceeded to study the ability of microglial cells differentiated from peripheral monocytes, referred to as *microglia-like cells* (MLCs), to integrate into the CNS. To generate MLCs, we followed a protocol from Carl Sellgren’s group [12], where they thoroughly characterized MLCs showing their higher similarity with microglia. Briefly, bone marrow cells collected from C57BL/6^GFP+^ mice were differentiated into MLCs in the presence of IL-34 and GM-CSF [12]. A comparative analysis of different microglial markers confirmed the differentiation of MLCs towards a microglia phenotype in our hands, when using primary microglia and monocyte-derived macrophages as reference: MLCs showed increased RNA and protein expression levels of the microglia specific markers TMEM119 and P2RY12 in comparison to macrophages (Additional file 1: Fig. S1). The expression of *Olfml3*, which is highly expressed in microglia, was comparable between MLCs and macrophages. However, the expression of *Adgre1*, a typical macrophage marker, was comparable between MLCs and microglia (Additional file 1: Fig. S1). Taken together, these data confirmed the differentiation of MLCs towards microglia characteristics as previously reported [12].

**Fig. 1:**
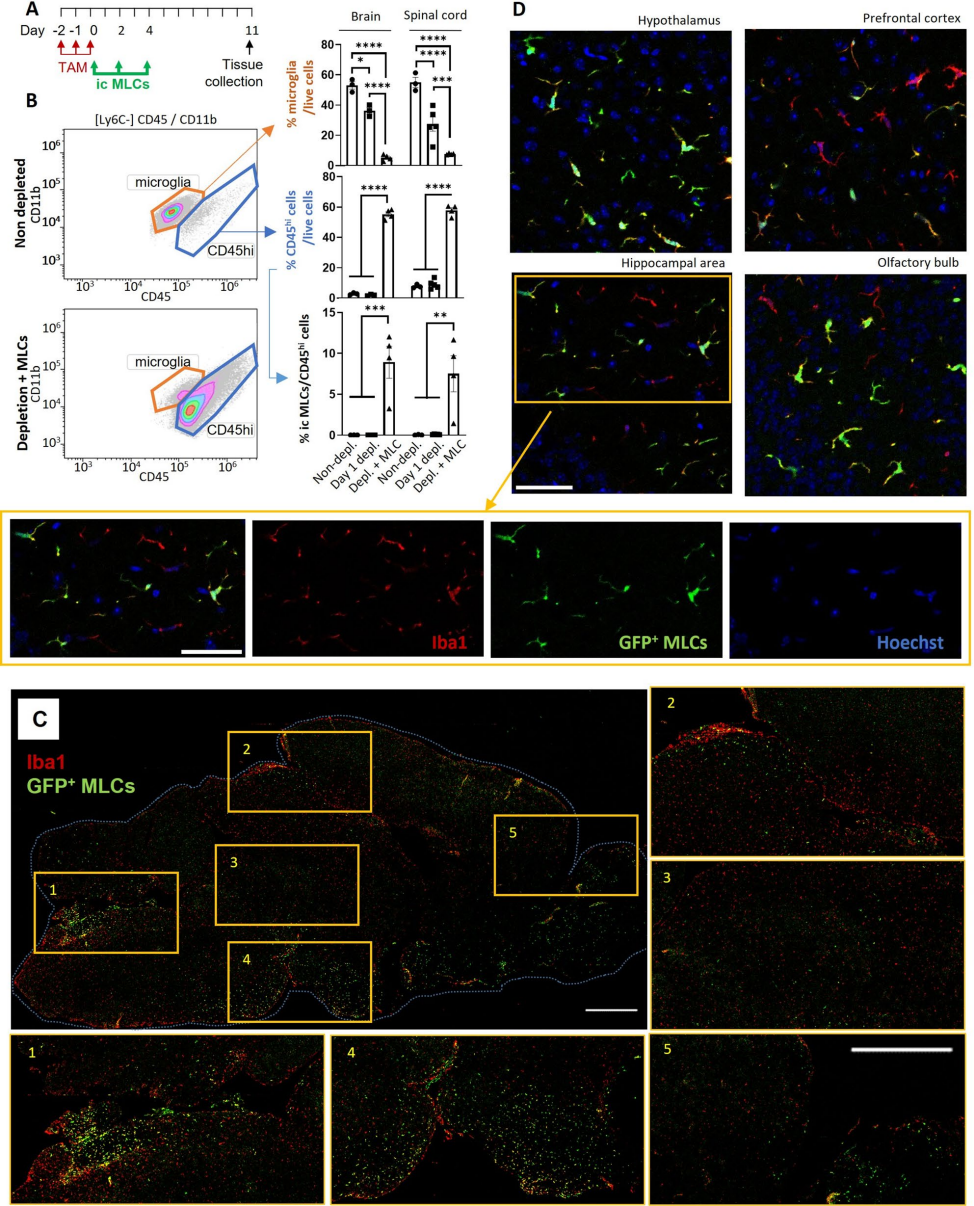
MLCs injected into cisterna magna are able to partially repopulate the brain parenchyma after microglia depletion. Cx3cr1^CreER^R26^DTA^ mice were treated with tamoxifen for 3 days and GFP^+^ MLCs administered via intracisterna (ic) 3 times each 48 h (**A**). 7 days after, repopulation of the microglial niche was monitored in one brain´s hemisphere and spinal cord by flow cytometry (**B**) and the other hemisphere by IHC (**C-D**). **A** Experimental design (Non-depl: *n* = 3; Day 1 depl: *n* = 5; Depl. + MLC: *n* = 4). **B** One brain´s hemisphere and spinal cord was analyzed by flow cytometry. Ic injected GFP^+^ MLCs gave rise to 7–9% of the repopulating population of the microglial niche. Graphs represent % cells and bars indicate mean ± SEM. **C** Representative image of microglia distribution (Iba1^+^) and magnification of 5 areas with enrichment of GFP^+^Iba1^+^ MLCs. Scale bar = 1 mm. **D** Microglial morphology detail of selected areas (green: injected GFP^+^MLCs, red: Iba1, blue: Hoechst). Scale bar = 50 μm

We proceeded to study the ability of MLCs to integrate into the CNS. Cx3cr1^CreER/-^R26^DTA/-^ mice were treated with tamoxifen 3 consecutive days to deplete the microglial niche [10] and GFP^+^ MLCs were administered i.c at days 0, 2 and 4 after the last tamoxifen injection (Fig. 1A). Hemibrains and spinal cords were processed for analysis of microglia repopulation by flow cytometry at day 11 post depletion. At this time point, endogenous microglia remains depleted (Additional file 1: Fig. S2) and repopulation by peripheral cells is already stablished as previously described by our group [10]. Briefly, myeloid cells were gated as CD11b^+^/CD45^+^ and monocytes excluded (Ly6C-). We identified endogenous microglia as CD11b^+^CD45^dim^ and repopulating cells as CD11b^+^CD45^hi^. Brains obtained from non-depleted animals and depleted animals (1 day post last tamoxifen injection, Day 1 depl.) were used as controls (Fig. 1B). Our analysis revealed that repopulating CD11b^+^CD45^hi^ cells constituted approximately 60% of the isolated live cells both in the brains and spinal cords at day 11 post microglia depletion (Fig. 1B). As expected, injected GFP^+^ MLCs were only present in the CD45^hi^ repopulating cell gate, representing around the 8% of the repopulating cells in both brain and spinal cord tissues (Fig. 1B).

Histological analysis of brain sections indicated an enrichment of the newly repopulating MLCs in areas with a high cerebrospinal fluid irrigation (Fig. 1C, areas 1, 2, 4 and 5), while still reaching more internal zones in the parenchyma (Fig. 1C, area 3). Higher magnification revealed the acquisition of microglial morphologies by GFP^+^ MLCs, similar to that observed for endogenously repopulating cells (Fig. 1D).

### MLCs maintain an intermediate signature distinct from endogenous microglia after integration in the brain parenchyma

**Fig. 2:**
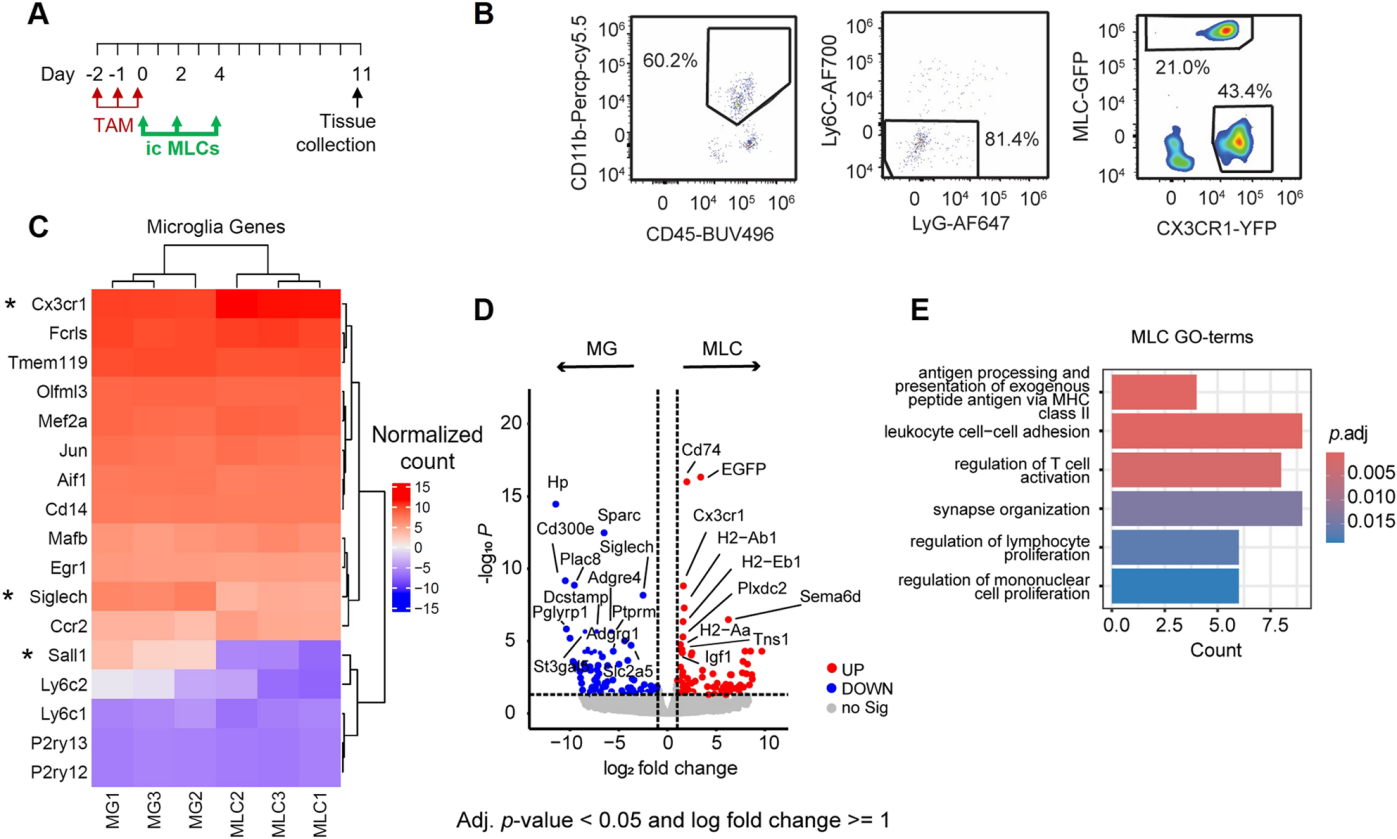
Comparative analysis of transcriptomic profiles between MLCs and endogenous microglia (MG). **A** Experimental design. **B** Gating strategy for cell sorting. **C** Heatmap for comparative expression of microglial genes. Genes with statistically significant differential expression (*Cx3cr1*, *Siglech* and *Sall1*) are indicated (*). **D** Volcano plot for differential expression between MLCs and microglia. **E** Gene Ontology (GO) analysis of DEGs in MLCs

Since MLCs partially express microglial signature genes in vitro (Additional file 1: Fig. S1), we next compared their transcriptomic profiles with those of resident microglia to determine the differences after adoptive transfer in vivo. We administered GFP^+^ MLCs intracisternally into microglia-depleted Cx3cr1^CreER − EYFP/−^R26^DTA/−^ recipient mice and sorted YFP^+^ endogenous microglia and GFP^+^ MLCs 11 days after the last tamoxifen injection for bulk transcriptomic analysis (Fig. 2A; gating strategy Fig. 2B). After QC, we detected 12,552 number of genes and intriguingly, only 147 genes were significantly different in MLCs compared to microglia (Fig. 2D; Additional file 2). When analysing specific microglial genes (Fig. 2C), only *Siglech* and *Sall1* were among the differentially expressed genes (Fig. 2D; Additional file 2), supporting the microglia-like identity of MLCs [18]. However, the lower expression of *Cx3cr1* in endogenous microglia could be a consequence of the host genetic background Cx3cr1^CreER − EYFP/−^ (Fig. 2C-D). Corroborating our in vitro findings (Additional file 1: Fig. S1), the relative expression of other microglia signature genes including *Olfml3*,* and Tmem119* [18] appeared to be slightly higher in microglia compared to in MLC, although these differences were not statistically significant (Fig. 2C). Besides, transcription factors involved in establishing microglia homeostatic functions and key in terminating developmental functions of pre-microglia towards adult microglia identity such as *Mef2a*, *Mafb* and *Jun* [10, 19] were expressed at comparable levels between MLCs and endogenous microglia, supporting the acquisition of microglia features by MLCs (Fig. 2C). In addition, *Slc2a5*,* Adrgr1*, and *St3gal5*, identified by us and others previously to be downregulated in repopulating monocyte-derived microglia [10, 20], were among the most down-regulated genes in MLCs (Fig. 2D). Moreover, we found MHC II genes to be among the most upregulated genes in MLCs (Fig. 2D), which is in accordance with low expression of MHC II on microglia [20]. This was also reflected in Gene Ontology (GO) analysis of DEGs in MLCs indicating enrichment in terms related to antigen processing and presentation, cell adhesion and regulation of T cell activation in MLCs (Fig. 2E). Taken together with previous reports, our findings demonstrate that, although partially distinct at transcriptional and functional level from microglia, adoptively transferred MLCs have the capacity to replace microglia and integrate in the brain parenchyma, acquiring a microglia-like signature, consistent with observations of other monocyte-derived microglia in previous studies [10, 20, 21].

### MLCs obtained from BALB/c and C3H mouse strains give rise to different anti-inflammatory phenotypes when subjected to an M2 differentiation protocol

Several mouse strains have been described to develop different immune reactions to the same stimulus (e.g. for C57BL/6, BALB/c and C3H [22]), something also observed when analysing cellular subsets such as macrophages [23, 24]. The genetic background influences the immune response to different stimuli and consequently plays a determinant role in disease outputs [23, 24]. Importantly, macrophage responsiveness and gene expression signatures observed in different mouse strains correlated with interindividual differences from human samples and served as a predictor for disease survival [24].

**Fig. 3:**
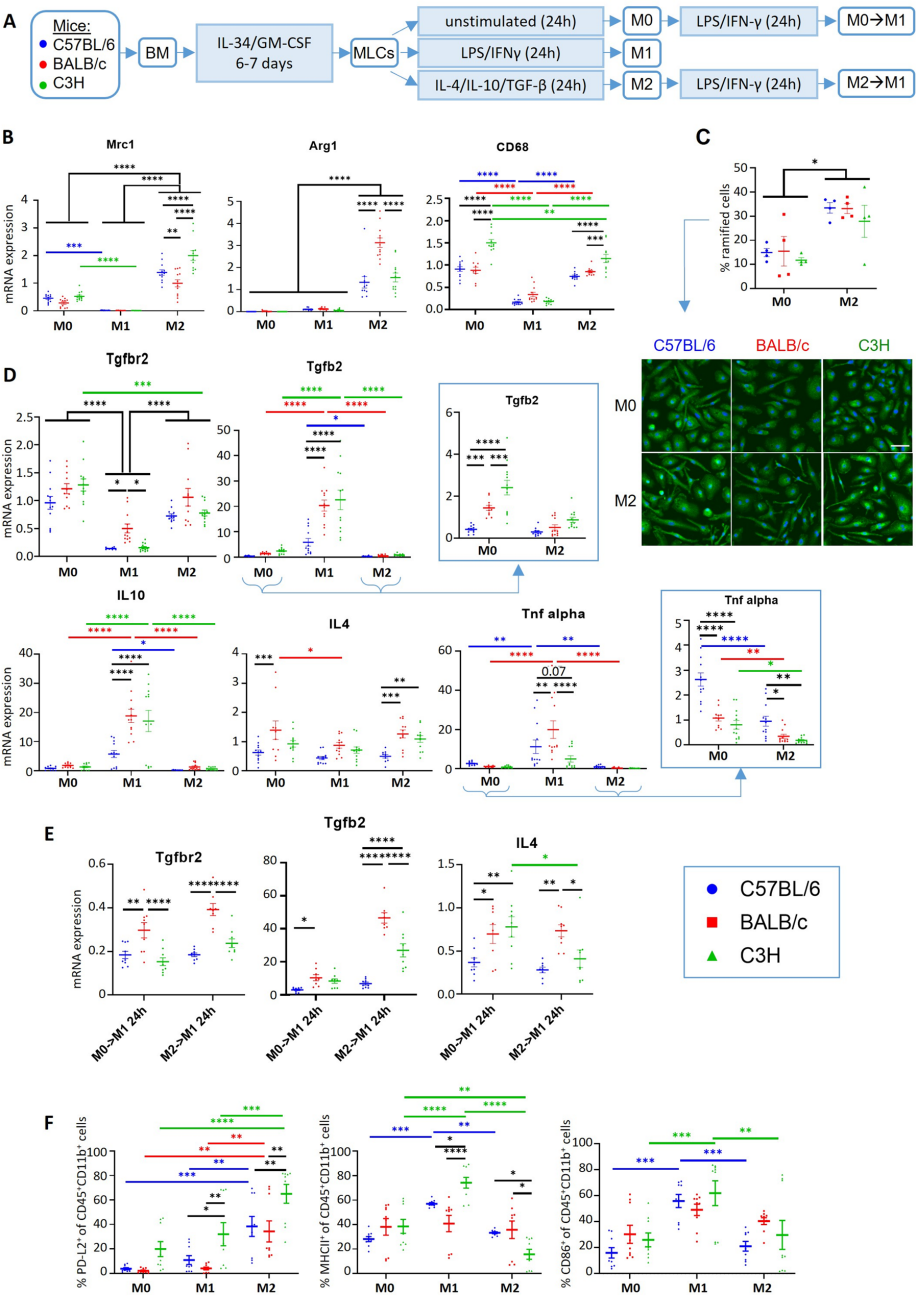
Immunophenotype characterization of C57BL6 (blue), BALB/c (red) and C3H/He (green) MLCs untreated (M0) or treated for 24 h with LPS/IFNg (M1) or Il-4, IL-10 and TGF-b (M2). **A** Experimental design. Resulting MLC phenotypes indicated as M0, M1, M2, M0◊M1 and M2◊M1. BM: Bone marrow. **B** mRNA expression levels of classical M1-M2 phenotypic markers. **C** M2 polarization of MLCs led to more ramified morphologies (green: IBA1, blue: Hoechst). Scale bar = 50 μm. Representative experiment (*n* = 4). **D-E** mRNA expression levels of cytokines and receptors following M0/M1/M2 polarization (**D**) and after a secondary proinflammatory stimulus (**E**). **F** % of CD45^+^CD11b^+^ cells positive for PD-L2, MHC II or CD86 measured by flow cytometry. Graphs represent data distribution as dots and bars indicating mean ± SEM. mRNA expression and flow cytometry data correspond to 3 independent experiments (*n* = 3)

Herein we selected the mouse strains BALB/c (H-2 ^d^) and C3H (H-2 ^k^), known for their milder immune response to some infections in comparison to C57BL/6 (H-2 ^b^) [23, 25]. We proceeded to characterize the immunophenotype of MLCs obtained from these three mouse strains to determine the therapeutic potential of using MLCs with different immunomodulatory properties (Fig. 3, Additional file 1: Fig. S3, Fig. S4). MLCs were differentiated from bone marrows and then treated with IL-10/IL-4/TGF-β to induce an anti-inflammatory phenotype (referred to as M2 for simplicity) and/or LPS/IFN-γ to study their response to a pro-inflammatory stimulus (denoted as M1) [26, 27] (Fig. 3A). We refer to MLCs in the unstimulated basal state as M0. Expression levels of different phenotypic markers were compared among polarization stages (M0, M1 and M2) and genotypic background (C57BL/6 [blue], BALB/c [red] and C3H [green]) (Fig. 3, Additional file 1: Fig. S3, Fig. S4).

The molecular characterization of immune receptors and cytokines revealed that both BALB/c and C3H MLCs expressed higher levels of some immunoregulatory markers with associated anti-inflammatory properties when compared to C57BL/6 MLCs in the basal state M0, but also after polarization with LPS/IFN-γ (M1) and/or IL-10/IL-4/TGF-β (M2). Specifically, the M2 polarization protocol with IL-4/IL-10/TGF-β led to an even more pronounced anti-inflammatory phenotype in both BALB/c and C3H MLCs compared to in C57BL/6 MLCs (Fig. 3). These M2 profiles were significantly different, e.g. expressing either higher levels of Arg1 (in the case of BALB/c MLCs) [23] or Mrc1 (C3H MLCs), both being phenotypic markers of anti-inflammatory myeloid cells (Fig. 3B). C3H MLCs also expressed higher levels of the endolysosomal protein CD68, considered a general myeloid marker, but that can indicate a specific anti-inflammatory profile [28]. In all cases polarizing MLCs with IL-10/IL-4/TGF-β led to phenotypic changes towards more ramified morphologies, characteristic of anti-inflammatory phenotypes (Fig. 3C).

When analysing cytokine signalling cascades, BALB/c and C3H MLCs exhibited lower levels of TNF-α expression in both basal state (M0) and after M2 polarization (Fig. 3D, Additional file 1: Fig. S4), and higher levels of the anti-inflammatory cytokines IL-4 (M2), IL-10 (M1) and TGF-β2 (Fig. 3D), the latter being especially evident after secondary stimulation with LPS/IFN-γ (Fig. 3E). Higher levels of the anti-inflammatory cytokine IL-10 were observed for M1 BALB/c and C3H MLCs after LPS/IFNg challenge (Fig. 3D), which points out a higher control of the immune response [29]. Importantly, BALB/c MLCs maintained higher expression levels of TGF-βR2, even after stimulation with LPS/IFN-γ, whose deficiency in myeloid cells is sufficient to induce fatal demyelinating disease [30] (Fig. 3D-E). The higher expression levels of TGF-βR2, TGF-β2 and IL-4 observed for M2 BALB/c MLCs were also maintained after a secondary LPS/IFN-γ stimulation (Fig. 3E), indicating the ability of M2-polarized BALB/c MLCs to partially maintain their anti-inflammatory phenotype even in a pro-inflammatory context (such as that encountered when transplanted into mice with neuroinflammation). The higher expression levels of IL-1β, TNFα and IL-6 by M2 BALB/c after secondary stimulation with LPS/IFN-γ (Additional file 1: Fig. S4), together with the Arg1 (Fig. 3B) and CD86 levels (Fig. 3F) and lower MRC1 (Fig. 3B) and IL-12 expression (Additional file 1: Fig. S3), also suggested enhanced tissue homeostasis functions [31, 32]. In contrast, increased expression of MRC1, CD68 (Fig. 3B) and TGF-β (Fig. 3D), and lower IL-12 levels (Additional file 1: Fig. S3) in C3H MLCs suggest a more debris scavenging and tissue healing functional phenotype [32]. Both BALB/c and C3H MLCs M2 phenotypes present immunoregulatory properties that could be beneficial in resolving neuroinflammation. A summary table of the variation in the expression levels of cytokines when comparing BALB/c or C3H/He MLCs with C57BL6 MLCs can be found in Additional file 1: Fig. S4D.

Flow cytometric analysis of membrane receptors revealed higher expression levels of PD-L2 (a suppressor of the immune response) and lower levels of the antigen-presenting molecule MHC II in M2 C3H MLCs (Fig. 3F), which could reduce the unleashed immune response when transplanted into MHC-mismatched mice and favouring engraftment. Interestingly, BALB/c MLCs did not upregulate MHC II or CD86 when challenged with LPS/IFN-γ (Fig. 3F), both markers being associated with antigen presentation and T cell activation [33], which could also reflect their resilience to immune responses.

The increased expression of IL-4 [34], TGF-β [10] and PD-L2 [35], along with lower levels of TNF-α [36] and MHC II [37, 38], observed in both BALB/c or C3H M2 MLCs indicated immunoregulatory properties that would potentially facilitate their immune acceptance in an MHC-mismatch context, but that could also have a therapeutic impact in autoimmune settings controlling the immune response.

Altogether, these data support the therapeutic potential of transplanting either BALB/c or C3H M2 MLCs, whose ability to regulate the immune response is supported by previous immunoregulatory characterizations of these mouse strains [23, 25, 39, 40].

### M2 MLCs differentiated from BALB/c and C3H mouse strains are tolerogenic *in vivo*

**Fig. 4:**
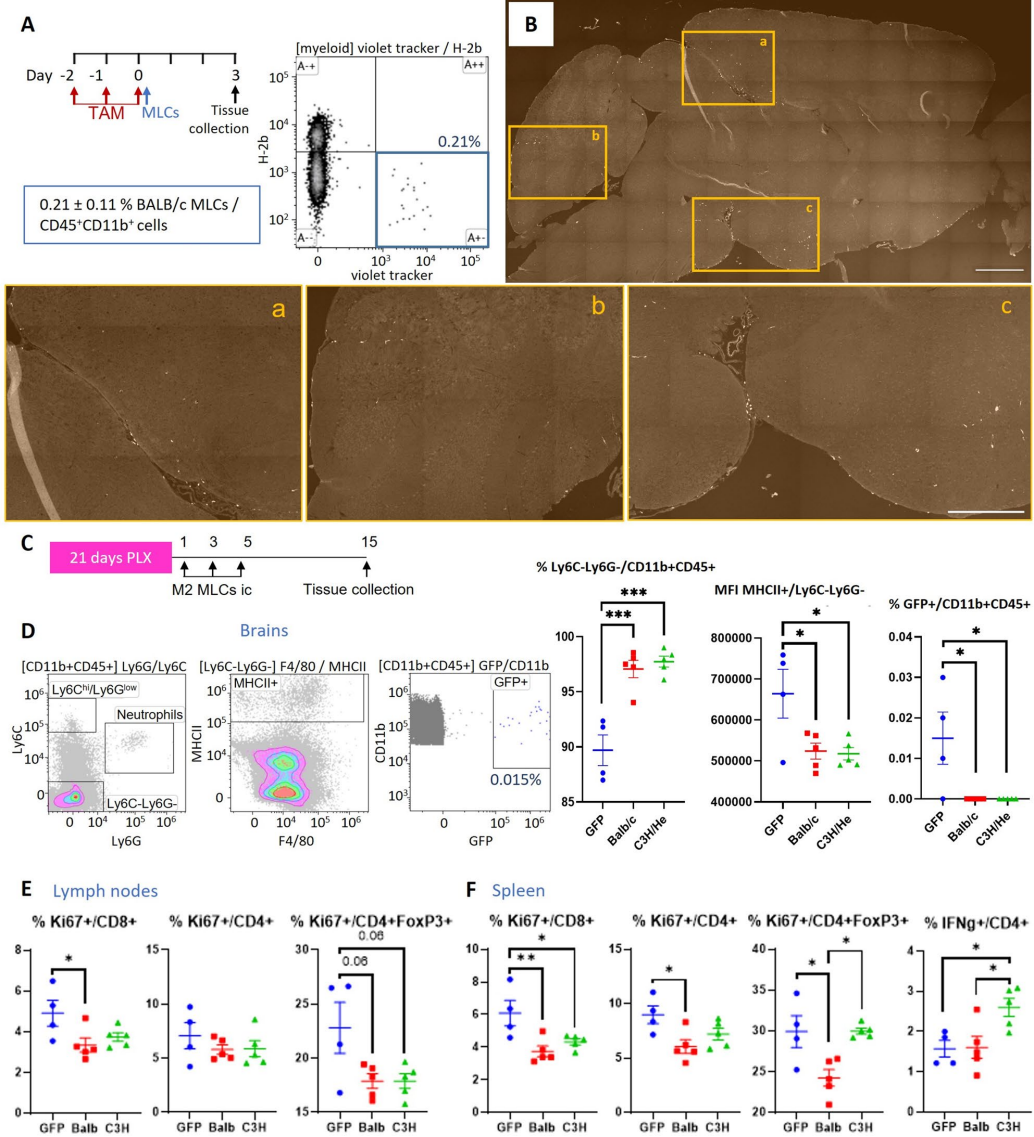
MLCs obtained from Balb/c and C3H present tolerogenic properties when injected in mice with a C57BL/6 background. **A-B** Cx3cr1^CreER^R26^DTA^ mice (C57BL/6 background) were treated with tamoxifen for 3 days and 5 10^5^ M2 BALB/c MLCs labelled with violet tracer were administered via intracisterna (ic) the last day of injection. 3 days later, MLCs were detected in the brain by both flow cytometry (**A**) and fluorescence microscopy (**B**) (*n* = 3). **B **Representative image of a brain section showing the integration of BALB/c MLCs (bright) into the brain of a mismatched mouse. Scale bar = 1 mm (magnifications a-b-c: Scale bar = 0.5 mm). **C-F** Mice with a C57BL/6 background received food containing PLX3397 for 21 days and, afterwards, M2 GFP-C57BL/6, BALB/c or C3H MLCs were administered ic 3 times each 48 h. 10 days later, brains (**D**), lymph nodes (**E**) and spleens (**F**) were analyzed by flow cytometry. Graphs represent data distribution as dots and bars indicating mean ± SEM (GFP-C57BL/6: *n* = 4; BALB/c: *n* = 5; C3H: *n* = 5)

The greater anti-inflammatory properties observed in vitro for M2 BALB/c and C3H MLCs led us to analyze the ability of MLCs to integrate into the brain in an MHC-mismatched background. Cx3cr1^CreER^R26^DTA^ mice (C57BL/6 background) were treated with tamoxifen for 3 consecutive days to eliminate microglia through the expression of diphtheria toxin in Cx3cr1^+^ cells, and 5 × 10^5^ M2 BALB/c MLCs pre-labelled with violet tracer were administered i.c. A single dose was sufficient to detect MLCs successfully integrated in an MHC-mismatched brain 3 days post-injection, as assessed using both flow cytometry (Fig. 4A) and fluorescence microscopy (Fig. 4B). The percentage of violet tracker^+^ cells (injected MLCs) were identified from the myeloid population (CD11b^+^ CD45^+^), and H-2b (H-2Kb, MHC-I) labelling was used as a confirmation of their origin (C57BL/6 H-2b^+^, BALB/c H-2b^−^, C3H H2-b^−^) (Fig. 4A.21 ± 0.11% BALB/c MLCs/CD45^+^CD11b^+^ cells). A similar result was observed for C3H and C57BL/6 MLCs (Additional file 1: Fig. S5). Analogous to what we observed in Fig. 1C, transplanted cells were predominately encountered in areas with high cerebrospinal fluid irrigation, where we could appreciate their entry site from ventricles and meninges and integration into the brain parenchyma at just 3 days post-injection of a single dose (Fig. 4B).

We next studied the host immune response to microglial repopulation with MHC-mismatched MLCs (Fig. 4C-F, Additional file 1: Fig. S6). For this purpose, we employed PLX3397 as a complementary microglia depletion approach to remove any immunomodulatory effect that tamoxifen could have in the Cx3cr1^CreER^R26^DTA^ mouse model [41]. Mice with a C57BL/6 background received food containing PLX3397 for 21 days to deplete the microglial niche. M2 C57BL/6^GFP+^, BALB/c or C3H MLCs were administered i.c each 48 h on 3 occasions. Ten days later brains (Fig. 4D), lymph nodes (Fig. 4E) and spleens (Fig. 4F) were analyzed by flow cytometry. GFP-labelled C57BL/6 MLCs were detected in the brain 10 days after the last MLC injection (Fig. 4D.015 ± 0.006 GFP^+^ MLCs/CD45^+^CD11b^+^ cells). Repopulation of the microglial niche after injection of MHC-mismatched MLCs led to an increased representation of the microglial fraction (Ly6G^−^Ly6C^−^) among the myeloid population (CD11b^+^CD45^+^) and, interestingly, to a lower expression of MHC II (Fig. 4D).

We also considered the possibility of having systemic immunomodulatory effects with our approach because of the intercommunication of the immune system between the CNS and the periphery, the possible disruption of the blood brain barrier (also observed in the EAE model), and as a precaution since we cannot discard a leakage of MLCs into the periphery due to technical limitations of the intracisternal injection procedure. Analysis of the lymphoid populations in lymph nodes (LN) and spleens revealed decreased levels of Ki67 in CD8^+^, CD4^+^ and/or CD4^+^FOXP3^+^ T cells in mice treated with MHC-mismatched MLCs, suggesting a lower T cell proliferation activity, especially in those receiving BALB/c MLCs (Fig. 4E-F). Splenic CD4^+^ T cells from mice injected with C3H MLCs also had higher expression of IFN-γ (Fig. 4F).

These data confirm the possibility of performing an allogenic transplant of M2 MLCs into the CNS, which importantly led to the tolerogenic reduction of MHC II levels in the brain and a slight reduction of T cell proliferation in the periphery.

### M2 MLCs differentiated from BALB/c and C3H are tolerogenic *in vitro*

Once we had confirmed the possibility of performing an allogeneic transplant of M2 MLCs into a healthy brain, we aimed to investigate their ability to resolve pathogenic insults. In a disease context, transplanted MLCs may encounter toxic debris and infiltrating immune cells in the CNS. We thus first performed in vitro assays to characterize their phagocytic ability and their immunoregulatory response to activated T cells.

**Fig. 5:**
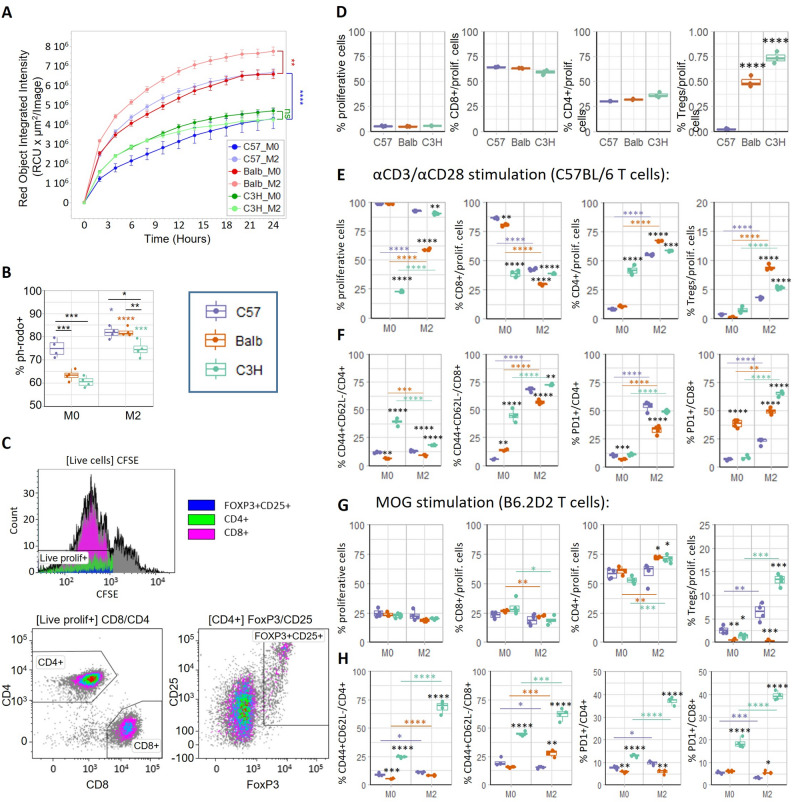
Functional in vitro assays. **A** MLCs were cultured for 24 h in 0.1% FBS media with 0.1 mg/ml zymosan-pHrodo and phagocytosis monitored by Live Cell Imaging (Incucyte). Significance (M0 vs. M2) AUC: One-way ANOVA (*n* = 10). **B** MLCs were cultured with 20ug/ml *E.Coli*-pHrodo in FBS-free DMEM/F12 and phagocytosis analysed by flow cytometry after 20 min (*n* = 4). **C-F **MLCs were cultured with CFSE-labelled T cells collected from C57 mice and unstimulated (**D**) stimulated with α-CD3/α-CD28 (**E**-**F**) for 72 h for subsequent analysis of T cell proliferation by flow cytometry (*n* = 4). **G-H **MLCs were co-cultured with T cells collected from B6.2D2 mice and stimulated with MOG(35–55) peptide for 72 h for subsequent analysis of T cell population by flow cytometry (*n* = 4). ANOVA, Tukey’s multiple comparison test. Data distribution (dots) is summarized as boxplots. Graphs represent a representative experiment of 3 (A: *n* = 10; B, D,E, G) or 2 (F, H) independent experiments

Phagocytosis of pH-rodo-labelled zymosan (Fig. 5A) or pH-rodo-labelled *E. coli* particles (Fig. 5B) was analyzed using Live Cell Imaging (Incucyte) or flow cytometry, respectively. We observed different phagocytic responses depending on the activation state. M2 polarization led to an increased phagocytic ability of MLCs derived from C57BL/6 and BALB/c mice, while it remained lower (Fig. 5B) or even unchanged (Fig. 5A) in C3H MLCs. Paradoxically, molecular characterization of C3H MLCs revealed the highest expression levels of the endolysosomal marker CD68 (Fig. 3B).

T cell proliferation assays were performed to study the potential immune response that MLCs could trigger when encountering infiltrating lymphocytes into the CNS in the context of EAE disease. To reflect the allogenic encounter, BALB/c or C3H MLCs were co-cultured with CSFE-labelled T cells obtained from C57BL/6 mice (Fig. 5C-F). C57BL/6 MLCs were used as control, mimicking a syngeneic situation.

After in vivo injection, MLCs would encounter T cells already activated by other endogenous antigen-presenting cells due to the EAE induction. To simulate this scenario and analyze the immunoregulatory effect of MLCs on already activated T cells, MLCs were co-cultured with T cells stimulated with αCD3/αCD28 for 72 h for subsequent analysis of T cell proliferation and immunophenotype by flow cytometry. Stimulation with αCD3/αCD28 led to high proliferation rates of T cells in the presence of M0 C57BL/6 or BALB/c MLCs (Fig. 5E). This proliferation was more moderate when MLCs were polarized to an M2 phenotype, especially in the case of BALB/c MLCs. Curiously, M0 C3H MLCs had the ability to drastically decrease the T cell response, but not when polarized to M2. The M2 polarization of C57BL/6 or BALB/c MLCs led to an increased proportion of CD4^+^ T cells, in comparison with the higher number of CD8^+^ cells obtained when unpolarized (M0) (Fig. 5E). However, C3H MLCs had the intrinsic ability (M0) to skew the T cell phenotype towards CD4^+^ cells, leading to a similar proportion (of CD4^+^ T cells) than that observed with M2 polarization of C57BL/6 or BALB/c MLCs. Both M2 BALB/c and C3H MLCs promoted the differentiation of T cells to Tregs after cell activation with αCD3/αCD28 (Fig. 5E). Notably, this increase in the proportion of Tregs was also observed in the absence of stimulation (Fig. 5D), highlighting the intrinsic ability of M2 BALB/c and C3H MLCs to promote immune tolerance in an MHC-mismatched context. As M2 C3H MLCs induced both higher levels of T cell activation (CD44^+^CD62L^−^) and PD1 expression (especially in CD8^+^ cells), this suggests the involvement of ongoing immune response exhaustion processes (Fig. 5F).

As a complementary approach, we analyzed the ability of MLCs to activate MOG-sensitive T cells. T cells derived from B6.2D2 mice (with a C57BL/6 background) were co-cultured with MLCs in the presence of MOG35-55 peptide. No differences were observed in the percentage of proliferative T cells, which remained at low levels among all groups indicating a low activation of T cells induced by MLCs (Fig. 5G). Aligned with the response observed with αCD3/αCD28 stimulation (Fig. 5E), MOG stimulation and co-culture with M2 BALB/c and C3H MLCs led to an increased proportion of CD4^+^ T cells, and M2 C3H MLCs led to higher levels of Tregs (Fig. 5G). However, Tregs levels with M2 BALB/c MLCs were lower instead (Fig. 5G). In accordance with αCD3/αCD28 stimulation (Fig. 5F), higher levels of T cell activation (CD44^+^CD62L^−^) and PD1 expression were also observed in the presence of M2 C3H MLCs and MOG stimulation (Fig. 5H). Proliferation data in Fig. 5E-H are also shown in Additional File 1: Fig. S7 organized by strain instead of treatment for clarity purposes.

These results support the therapeutic potential of M2 BALB/c MLCs specifically through their phagocytic ability, whereas their ability to induce regulatory T cells depended on the experimental setting (non-stimulated, αCD3/αCD28 or MOG stimulation). Meanwhile, M2 C3H MLCs could induce both regulatory T cells and other immune tolerance mechanisms through enhancing the expression of PD-1 in T cells, consistent with the elevated levels of its ligand PD-L2 detected in C3H MLCs (Fig. 3F). These phenotypic changes suggest ongoing tolerogenic exhaustion or anergy processes, maybe in part due to the higher activation of T cells (CD44^+^CD62L^−^).

Taken together with the molecular characterization of MLCs (Fig. 3) and the reduced levels of MHC II in the endogenous repopulating microglia following adoptive transfer of MHC-mismatched MLCs (Fig. 4D), these data support the potential benefit that selected M2 MLCs could promote in an MHC-mismatched context for the treatment of autoimmune diseases such as MS.

### Intra-cisternal administration of M2 MHC-mismatched MLCs to microglia-depleted C57BL/6 mice enhances immune tolerance mechanisms and reduces EAE severity

Due to the tolerogenic potential that both BALB/c and C3H M2 MLCs exhibited in the previous experiments, we wanted to determine the therapeutic impact that their transplantation could have in the EAE model of MS. We hypothesized that immune tolerance mechanisms enhanced by the allogeneic transplantation of M2 MHC-mismatched MLCs in the CNS could yield therapeutic effects in autoimmunity. As women are about 2–3 times more likely to develop MS than are men [6], we decided to perform this study in female mice [42].

We induced the EAE MS model in Cx3cr1^CreER/−^R26^DTA/−^ female mice with a C57BL/6 (H-2b) genetic background. At the onset of symptoms mice were treated with tamoxifen (days 9–11 after EAE induction) followed by the i.c administration of PBS or M2 MLCs on days 11, 13 and 15. Mice were distributed into 5 experimental groups:

i) Syngeneic replacement (H-2b→H-2b): Cx3cr1CreER/-R26DTA/- (H-2b) + tamoxifen + C57BL/6GFP+ (H-2b) MLCs
ii) Allogeneic replacement (H-2d→H-2b): Cx3cr1CreER/-R26DTA/- (H-2b) mice + tamoxifen + BALB/c (H-2d) MLCs
iii) Allogeneic replacement (H-2k→H-2b): Cx3cr1CreER/-R26DTA/- (H-2b) mice + tamoxifen + C3H (H-2k) MLCs
iv) Endogenous repopulation: Cx3cr1CreER/-R26DTA/- (H-2b) mice + tamoxifen + PBS
v. Non-substitution of the microglial niche: C57BL/6 mice + tamoxifen + PBS.

**Fig. 6:**
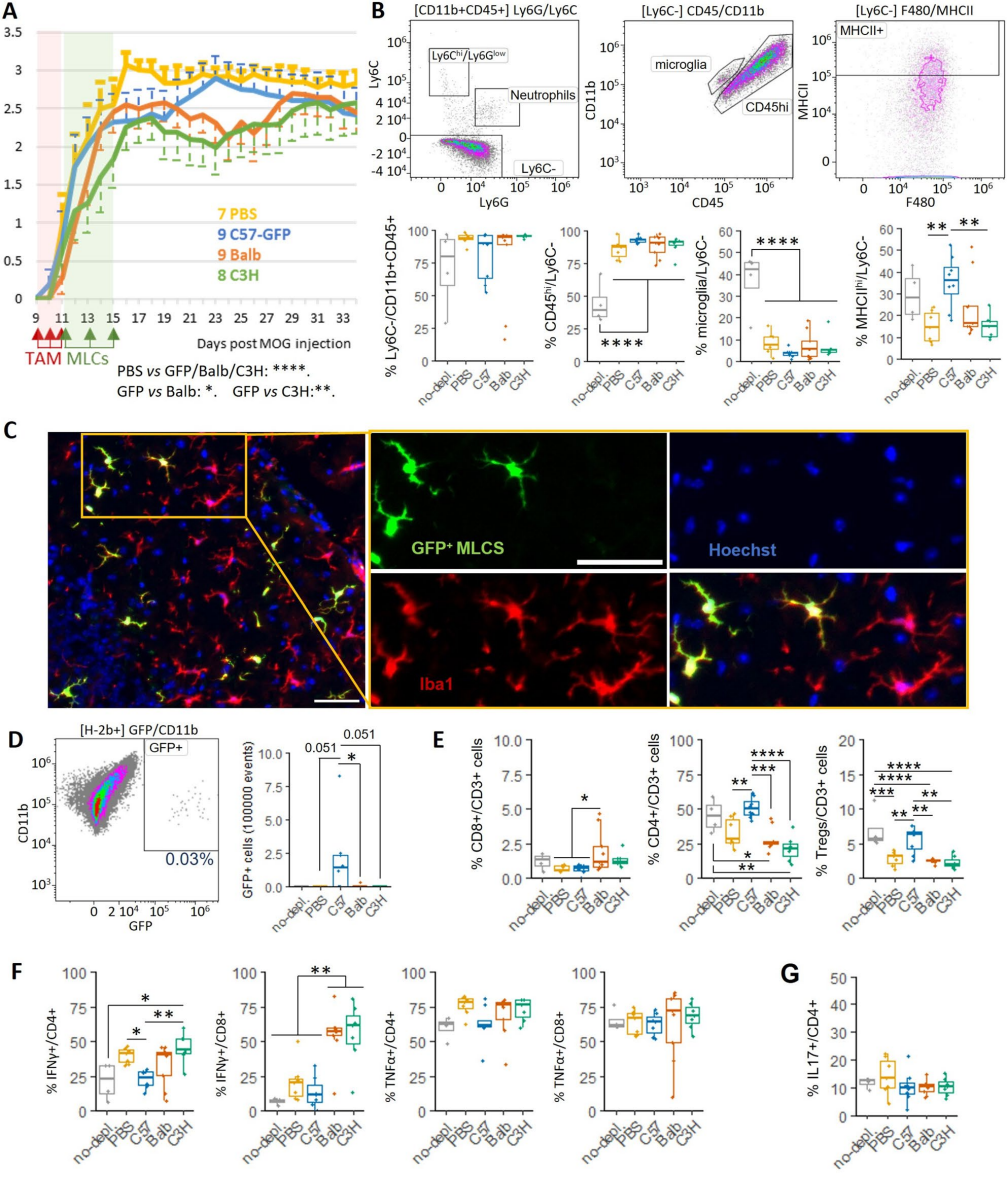
Long-term analysis at day 34 of microglia depletion and MLC injection in EAE mice: 4-to-8 months old Cx3cr1^CreER^R26^DTA^ mice (C57BL/6 background) were treated with tamoxifen for 3 days at the onset of EAE, and M2 MLCs or PBS were administered ic 3 times each 48 h. Mice were sacrificed for analysis at day 34 post-EAE induction. **A** Scoring of the progression of the EAE of mice receiving ic PBS (yellow, *n* = 7), GFP-C57BL/6 (blue, *n* = 9), BALB/c (orange, *n* = 9) or C3H (green, *n* = 8) MLCs. Repeated Measures One-way ANOVA (*p* < 0.0001): PBS vs. GFP/Balb/C3H: ****; GFP vs. Balb: *; GFP vs. C3H**. **B**,** D-F** Flow cytometry analysis of the spinal cords at the endpoint of the EAE, in comparison with EAE-induced C57BL/6 mice (treated with tamoxifen ip and PBS ic) as a non-depleted control. **B** Myeloid composition. **C-D** GFP expression in C57BL/6 MLCs allowed to confirm the presence of engrafted cells at the endpoint of the EAE by flow cytometry (**D**) and immunofluorescence (**C**) (green: GFP, red: Iba1, blue: Hoechst; brain tissue, Scale bar = 50 μm). **E** Infiltration into the spinal cord of lymphocytes. **F-G** Cytokine expression profile of the infiltrated CD4^+^ and CD8^+^ cells. One-Way ANOVA. Data distribution (dots) summarized as boxplots

First, we performed a pilot experiment in 4- and 8-month-old females in which we monitored EAE disease progression for 34 days and collected spinal cords for further analysis (Fig. 6, Additional file 1: Fig. S8). This experiment revealed a transient and significant amelioration of EAE disease progression around days 21–27 when treated with MHC-mismatched MLCs (allogeneic replacement) in comparison with those mice treated with either PBS (endogenous repopulation) or GFP^+^ C57BL/6 MLCs (syngeneic replacement) (Fig. 6A). While the amelioration of EAE disease progression appeared more evident and long-lasting in 4-month-old mice, the number of mice included in this pilot experiment did not allow us to draw conclusions based on age (data analysis not included).

GFP^+^ labeling of C57BL/6 MLCs allowed us to confirm their engraftment into the CNS and adoption of a microglial morphology at day 34 after EAE induction (Fig. 6C). Spinal cords, the main region affected in the EAE model, were processed for analysis of immunoregulatory cells by flow cytometry. Consistent with previous data [10], depletion of the microglial niche led to a decrease in endogenous microglial numbers and an increase in CD45^hi^ repopulating cells (Fig. 6B). In comparison with those mice receiving syngeneic GFP^+^ C57BL/6 MLCs, C3H MLCs administration reduced the MHC II levels in the microglial population, and a similar tendency (although not significant) was also observed for mice treated with BALB/c MLCs. While BALB/c and C3H MLCs were not detected due to the limited specificity of the tested antibodies, GFP^+^ labeling of C57BL/6 MLCs allowed us to confirm the engraftment of MLCs into the spinal cord by flow cytometry (Fig. 6. D).

Despite the transient therapeutic amelioration of the EAE observed, analysis of the lymphocytic populations at day 34 showed lower levels of infiltrating CD4^+^ T cells and Tregs in spinal cords from mice receiving MHC-mismatched MLCs in comparison with C57BL/6^GFP+^ recipients (Fig. 6E). Higher proportions of CD4^+^IFN-γ^+^ T cells in C3H MLCs recipients and CD8^+^IFN-γ^+^ T cells in BALB/c and C3H recipients were also observed (Fig. 6F). No differences were detected in the expression levels of T cell-derived TNFα (Fig. 6F), IL-17 (Fig. 6G), IL-10 or of the proliferation marker Ki67 (Additional file 1: Fig. S8C-D) in T cells among the groups. These results prompted us to consider the possible participation of other tolerogenic mechanisms.

**Fig. 7:**
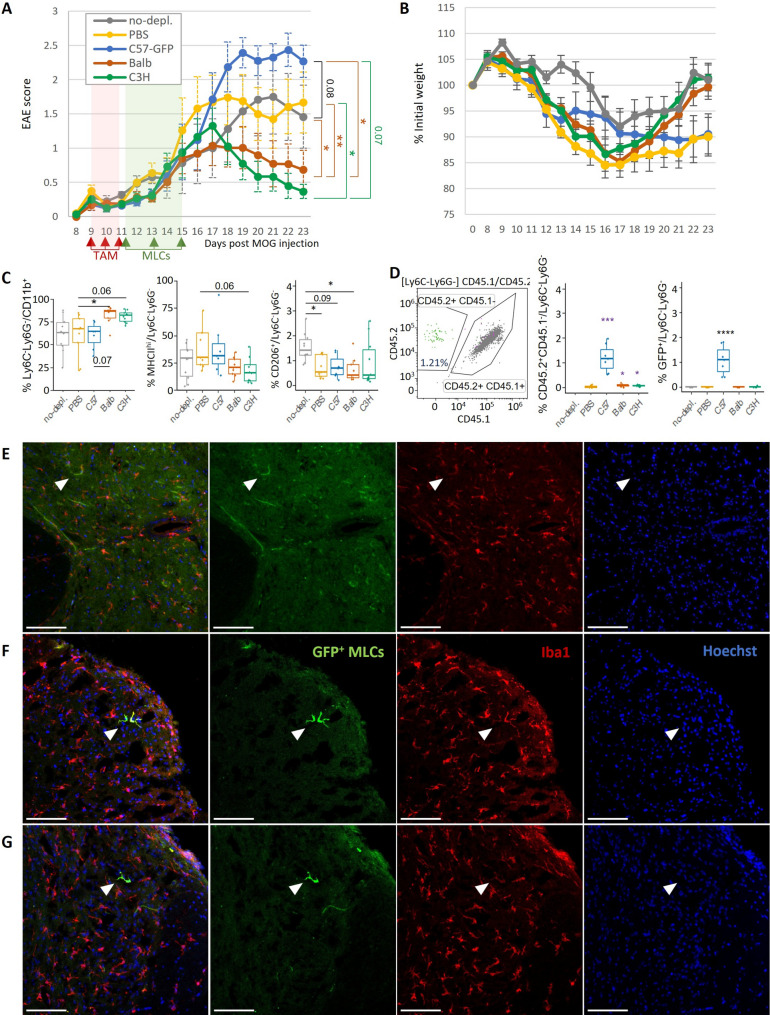
Short-term analysis at day 23 of microglia depletion and MLC injection in EAE mice: 4-month-old CD45.1/CD45.2 Cx3cr1^CreER^R26^DTA^ and C57BL/6 mice were treated with tamoxifen for 3 days at the onset of EAE symptoms. Microglia-depleted Cx3cr1^CreER^R26^DTA^ mice received a total number of 10^6^ M2 MLCs (or PBS) administered in 3 ic injections: PBS (yellow, *n* = 9), GFP-C57BL/6 (blue, *n* = 8), BALB/c (orange, *n* = 10) or C3H (green, *n* = 10). C57BL/6 mice received PBS as negative control of microglia depletion (gray, *n* = 10). Mice were sacrificed for analysis at day 23 post-EAE induction. **A** Scoring of mice developing EAE. Repeated Measures One-way ANOVA (*p* < 0.01). **B** % weight compared to the initial weight at the day of induction of EAE (Day 0). Daily values represented as mean ± SEM. **C-D** Flow cytometry analysis of spinal cords at the endpoint of the EAE (day 23). **C** Myeloid cell composition. One-way ANOVA. **D** Identification of injected MLCs in microglia-depleted mice was detected as CD45.2^+/+^ (CD45.1^−^) cells (Student’s one-tailed unpaired t test vs. PBS group) and confirmed with GFP labelling for C57^GFP+^ MLCs (colored in green in the dot plot). **E-G** Immunofluorescence of spinal cord sections from GFP^+^MLCs recipients showed the integration of GFP^+^Iba1^+^ MLCs (white arrows) in both grey matter (**E**) and white matter (**F**,** G**) areas (green: GFP, red: Iba1, blue: Hoechst; Scale bar = 100 μm)

**Fig. 8:**
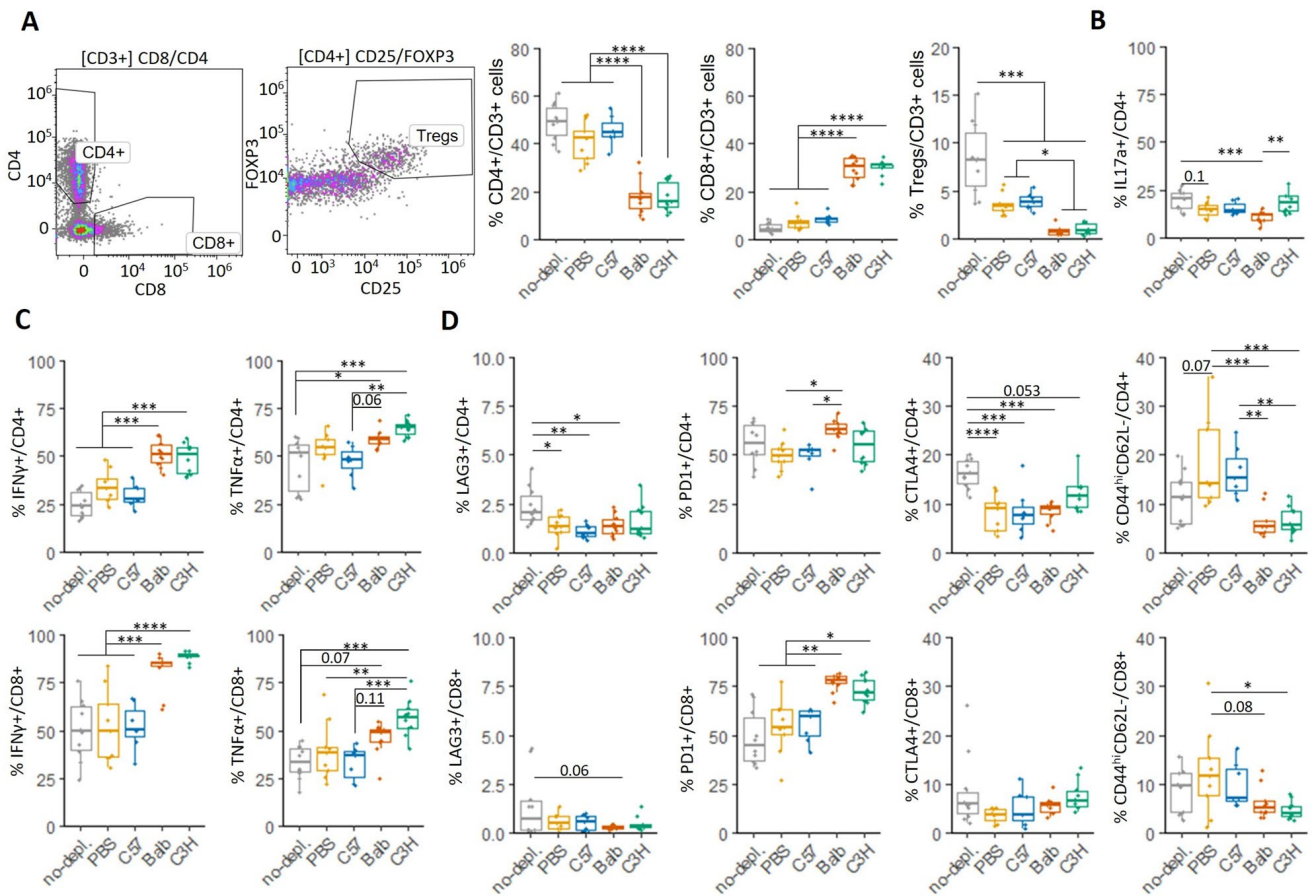
Flow cytometry analysis of infiltrated T cells into the spinal cord at day 23 post- EAE induction of microglia-depleted and MLC-injected mice (continuation of Fig. 7). **A** % of CD4^+^, CD8^+^ and Tregs within infiltrated T cells (CD3^+^). **B-C** % of CD4^+^ or CD8^+^ T cells positive for different cytokines. **D** Activation profile of CD4^+^ and CD8^+^ T cells according to the expression of different receptors. One-way ANOVA. Data distribution (dots) summarized as boxplots

To decipher the mechanisms that could be involved in this therapeutic effect around days 21–27 for MHC-mismatched MLCs, another analogous experiment was performed in 4-month-old CD45.1/CD45.2 Cx3cr1^CreER/−^R26^DTA/−^ female mice and cellular populations were analyzed at day 23 post-EAE induction (Figs. 7 and 8), a timepoint at which a greater amelioration of EAE was observed in both independent experiments (Figs. 6A and 7A). We used recipient mice with CD45.1/CD45.2 genetic background to enable detection of the injected MLCs (CD45.2/CD45.2). Spinal cords were processed for the examination of immunoregulatory cells by flow cytometry.

Analysis of spinal cords after exclusion of monocytes and neutrophils at day 23 revealed an increase in the proportion of Ly6C^−^Ly6G^−^ myeloid cells, which we refer to as the microglial population, in the spinal cords of the mice treated with MHC-mismatched MLCs (Fig. 7C; gating strategy Fig. S9A). We confirmed the CD45^+^ isoform signal of microglial populations, which as expected indicated CD45.2 positivity in the injected cells and CD45.2/CD45.1 positivity in the endogenous cells (Fig. 7C; Additional file 1: Fig. S9A). Although not statistically significant, the administration of C3H and BALB/c MLCs seemed to promote a reduction in the MHC II levels in the microglial population (Fig. 7C) similar to that observed at day 34 after EAE induction (Fig. 6B). The increase in the microglial proportion (Ly6C^−^Ly6G^−^) and reduction of MHC II levels in mice treated with MHC-mismatched MLCs were also both observed after depletion with PLX (Fig. 4D).

We next used the CD45.1/CD45.2 labelling in flow cytometry to distinguish injected BALB/c and C3H MLCs (CD45.2/CD45.2) from endogenous microglial cells (CD45.1/CD45.2) in spinal cords collected at day 23 (Fig. 7D). GFP^+^ labeling of C57BL/6 MLCs allowed us to validate the identification of injected MLCs according to CD45.1/CD45.2 antibodies, as all injected GFP^+^ cells (colored in green) appeared in the CD45.2^+^CD45.1^−^ gate (Fig. 7D). While percentages of C57BL/6^GFP+^ MLCs (mean ± SEM = 1.21 ± 0.21%; syngeneic for H-2b) were significantly higher, allogeneic BALB/c (0.10 ± 0.02%) and C3H (0.09 ± 0.01%) MLCs were also detectable at lower levels (Fig. 7D). As expected, all endogenous microglia in the non-depleted group were CD45.2^+^CD45.1^−^ due to the C57BL/6 genetic background (data not included in the graph for clarity purposes, Fig. 7D).

We further validated the presence of injected GFP^+^ MLCs in the spinal cord by immunofluorescence. MLCs appeared to have adopted microglia-like morphology and were present in both grey (Fig. 7E) and white matter (Fig. 7F-G). MLCs were distributed in low numbers in meningeal surfaces and in the parenchyma at both lesion sites and normal tissue. In accordance with the lower EAE scores observed for MHC-mismatched MLCs recipients, the presence of small lesions was especially evident in spinal cords from mice receiving C57BL/6^GFP+^ MLCs (Additional file 1: Fig. S10).

While Cx3cr1 expression made BBB-associated macrophages (BAMs, CD206^+^) susceptible to tamoxifen [43], no differences were observed between microglia-depleted groups at day 23 (Fig. 7C, third panel). Neither were significant changes observed in the proportion of monocytes or neutrophils among groups at day 23 (Additional file 1: Fig. S9A) or at day 34 after EAE induction (Fig. 6B; Additional file 1: Fig. S8A).

We also analyzed the lymphocytic infiltration into the spinal cord at day 23 (Fig. 8; Additional file 1: Fig. S9B-G). No differences were evident for total CD3^+^ T cell or CD19^+^ B cell numbers (Additional file 1: Fig. S9B-D). However, mice receiving MHC-mismatched MLCs had a higher proportion of CD8^+^ T cells (Fig. 8A). Elimination of the microglial niche led to lower levels of Tregs, these being even lower in those mice receiving BALB/c or C3H MLCs (Fig. 8A). CD4^+^ T cells from mice receiving BALB/c MLCs expressed lower levels of IL-17a (Fig. 8B). In contrast, BALB/c and C3H MLCs recipients had a higher proportion of CD4^+^IFN-γ^+^ and CD8^+^IFN-γ^+^ T cells, whereas C3H MLCs recipients also showed significant higher levels of CD4^+^TNF-α^+^ and CD8^+^TNF-α^+^ T cells (Fig. 8C). No differences were detected in the expression levels of IL-10 or Ki67 (Additional file 1: Fig. S9E-F).

When comparing the lymphocytic infiltration in the short-term experiment (day 23, Fig. 8) with that observed in the long-term experiment (day 34, Fig. 6), we could appreciate the maintenance of the lower levels of infiltrated CD4^+^ T cells and Tregs in spinal cords from mice receiving MHC-mismatched MLCs (Figs. 6E and 8A). While the increased TNF-α levels at day 23 had already dissipated at day 34, higher proportions of CD4^+^IFN-γ^+^ T cells in C3H MLCs recipients and CD8^+^IFN-γ^+^ T cells in BALB/c and C3H recipients were still observed (Figs. 6F and 8C). Importantly, long-term analysis of EAE at day 34 revealed very low levels of CD8^+^ T cells among all groups (Fig. 6E), indicating that the higher levels observed at the peak of the disease at day 23 in mice receiving MHC-mismatched MLCs (Fig. 8A) constitutes a transient effect.

Finally, we wanted to determine which tolerance mechanisms could be participating in controlling the immune response in the CNS that could explain the therapeutic effect observed around day 23. Since a Treg response was not detected as possible mechanism either in the pilot experiment at day 34 (Fig. 6E) or at day 23 (Fig. 8A), we decided to analyze other inhibitor receptors of T cell activity. Microglial depletion led to a decreased expression of LAG3 and CTLA4 in CD4^+^ T cells (Fig. 8D). However, mice treated with BALB/c MLCs had higher proportions of CD4^+^PD1^+^ and CD8^+^PD1^+^ T cell populations (Fig. 8D). Mice treated with C3H MLCs presented more CD8^+^PD1^+^ T cells (Fig. 8D) and a higher proportion of CD4^+^CTLA4^+^PD1^−^ T cells (Additional file 1: Fig. S9G). Mice receiving BALB/c or C3H MLCs also had a lower proportion of activated T cells (CD44^hi^CD62L^−^) (Fig. 8D).

Taken together, these data support a greater control of the lymphocytic response through immune tolerance processes triggered by the adoptive transfer of MHC-mismatched anti-inflammatory MLCs. Although the increase in Tregs obtained in T cell proliferation assays (Fig. 5C-E) was not observed, other detected tolerogenic mechanisms could explain the therapeutic effect in EAE. Analysis of CNS-infiltrated T cells revealed lower levels of activation (CD44^hi^CD62L^−^) as well as an increase in receptors involved in T cell functional inactivation or anergy (CTLA-4, PD-1). Increased production of IFN-γ has been associated with protective effects in EAE and MS and could be involved in the lower levels of activated T cells [44, 45]. Likewise, the lower levels of MHC II and IL-17a could also contribute to the protective effects observed in mice receiving C3H and BALB/c MLCs, respectively.

Our data support the promotion of anergy and exhaustion processes as tolerance mechanisms which could explain the therapeutic effect observed when administering MHC-mismatched MLCs in the autoimmune disease model EAE of MS.

## Discussion

Pre-clinical studies in mice systematically ignore the impact that different genetic backgrounds can have in molecular and functional characterizations and the success of immunotherapeutic strategies [24]. In this study we took into account the inter-individual genetic and phenotypic heterogeneity to first evaluate MLCs responses to pro- and anti-inflammatory stimuli. The use of different polarization stages and acquisition of functional abilities by cells obtained from MHC-mismatched mice highlight the importance of confirming a specific protocol in genetic backgrounds other than C57BL/6. Choosing the most suitable profile is therefore essential for succeeding in the design of cell therapy strategies and sets the path to personalized clinical approaches [24].

We compared MLCs obtained from C57BL/6 mice with MLCs derived from BALB/c and C3H mice, two strains known for their milder immune response to some infections [23, 25]. While our polarization protocol led to similar morphological changes, molecular characterization revealed different transcriptional signatures both in basal conditions (M0) and in response to pro- (M1) and anti-inflammatory (M2) stimuli. We referred to our different polarization protocols using the common M0-M1-M2 nomenclature just for simplifying the narrative, without any intention of narrowing the complexity and versatility of microglial activation to just 3 phenotypic stages. MLCs obtained from BALB/c and C3H mice exhibited more anti-inflammatory phenotypes in comparison with C57BL/6. These phenotypes were quite different, encompassing features that could promote beneficial outcomes when transplanted to EAE mice in an MHC-mismatched context, such as increased expression of IL-4 [34, 46], TGF-β [30], and PD-L2 [35], along with lower levels of TNF-α [36]and MHC II [37, 38].

TGF-β signaling plays a critical role in preventing myeloid-mediated CNS pathologies [30, 47]. The upregulation or TGF-β family members observed in C3H and BALB/c MLCs could enhance the maintenance of homeostatic microglia gene signatures but also enhance tolerogenic mechanisms such as Treg differentiation. TGF-β2, more highly expressed in C3H and BALB/c MLCs, is known to reduce severity in both Theiler’s virus-induced encephalitis [48] and in EAE [49] mouse models. The higher levels of TGF-βR2 expression observed in BALB/c MLCs could be involved in controlling the expression of the antigen presenting molecule MHC II [30], the expression of which remained at low levels after LPS/IFN-γ challenge. Increased levels of IL-4, mainly observed in BALB/c MLCs, could also contribute to the observed ameliorated progression of EAE [46] and prevent allograft rejection [34].

Interestingly, the lower levels of MHC II observed in M2 C3H MLCs seemed to propagate to the endogenous microglial population when transplanted into C57BL/6 mice (also with M2 BALB/c MLCs) and after EAE induction. C3H MLCs also expressed higher levels of PD-L2 which could also play a role in promoting immune tolerance through anergy or exhaustion mechanisms in T cells [35]. While both PD-L1 and PD-L2 have been associated with protective effects in MS [35], previous data in our group indicated a more pronounced upregulation of PD-L1 after polarizing macrophages to an M1 phenotype [27]. Our M2 polarization protocol led to a specific upregulation of PD-L2 expression, which was even retained after a secondary stimulation with LPS/IFN-γ [27], constituting a valuable marker for our tolerogenic phenotype.

 In vitro functional assays illustrated differences in the intrinsic phagocytic ability of MLCs depending on the activation state. While C57BL/6 and BALB/c MLCs significantly increased their phagocytic activity in response to an M2 polarization, a more subtle effect (or none) was observed for C3H MLCs. Paradoxically, C3H MLCs had the highest expression levels of the endolysosomal marker CD68, which in contrast could indicate a specific anti-inflammatory profile together with the upregulation of TGF-β [28].

Altogether, these data support the therapeutic potential of transplanting either BALB/c or C3H M2 MLCs, whose ability to regulate the immune response may facilitate their engraftment and have a beneficial effect to resolve neuroinflammation and promote tolerogenic mechanisms in autoimmune settings.

We therefore wanted to test the therapeutic potential of administering M2 MHC-mismatched MLCs after depleting the microglia niche in the autoimmune disease model EAE. We first optimized a protocol for the administration of MLCs directly into the brain via the intra cisterna magna after microglial depletion. A partial repopulation of the microglial niche was achieved, being especially evident in those areas with higher cerebrospinal fluid irrigation. Of note, while the i.c administration route has previously been shown to efficiently release small particles (such as fluorescent tracers and virus) into the brain [50, 51], the adoptive transfer of cells resulted in no [52, 53] or very modest penetration into the parenchyma [54–56]. To our knowledge, our study is the first demonstration that i.c administration of cells leads to a significant penetration into the brain parenchyma and highlights the necessity of emptying the niche for their integration.

Importantly, we could also confirm the integration of MHC-mismatched MLCs into the brain by pre-labelling with violet tracer prior to their injection. While violet tracer labelling allowed us to successfully distinguish MHC-mismatched MLCs from endogenous myeloid cells 3 days post-administration, we could not assess their long-term occupancy due to the limited stability of the dye. Therefore, we had to perform just one injection of MLCs, instead of 3 injections every other day, which could have affected the numbers of MLCs detected in the brain in comparison with other experiments.

When comparing the percentage of engraftment of GFP^+^ MLCs between different experimental settings, repopulation efficiencies were higher in non-disease Cx3cr1^CreER/−^R26^DTA/−^ mice (Figs. 1 and 2) than in those mice subjected to EAE induction (Figs. 6 and 7). Depletion of microglia with PLX3397 also led to a lower engraftment efficiency (Fig. 4D), which could be a consequence of different endogenous repopulation dynamics or the presence of remaining CSF1R inhibitor affecting the first MLC injections. However, the similarities observed among microglia depletion strategies (genetic or pharmacological), such as the lower MHC II expression in endogenous microglia in BALB/c or C3H MLCs recipients or the increase of IFN-γ^+^CD4^+^ cells in C3H MLCs recipients, strengthen the conclusion that the effects are attributable to the MLC treatment, independent of the depletion model used.

We selected female mice for our study in EAE due to the higher incidence of MS in women [6] and previous results from our group [42]. Besides, previous data from the group demonstrated that Cx3cr1^CreER^R26^DTA^ and C57BL/6 female wild type mice exhibit identical clinical score profiles and cumulative EAE scores [42]. This allowed us to use C57BL/6 mice as an additional ‘non-substitution of the microglial niche’ control group for tamoxifen treatment and i.c injection (with PBS) procedure, while allocating all the available Cx3cr1^CreER/−^R26^DTA/−^ mice for the microglia depleted groups. According to previous data [10], depletion of the microglial niche led to a decrease in endogenous microglial numbers and an increase in CD45^hi^ repopulating cells (Fig. 6B). While GFP labeling allowed the detection of transplanted isogenic C57BL/6^GFP+^ MLCs, a CD45.1/CD45.2 genetic background was necessary for a reliable detection of BALB/c and C3H MLCs (CD45.2/CD45.2). Anti-H2^d^ and -H2^K^ antibodies were also tested to distinguish BALB/c and C3H/He MLCs in recipient mice, although their specificity was not conclusive (data not included). Our results confirmed a modest integration of allogeneic and syngeneic (at higher levels) MLCs into the brain. GFP labelling also confirmed the adoption of a microglial morphology.

When analyzing the population of CNS-infiltrated T cells, increased levels of PD-1 (and CTLA-4) were observed after adoptive transfer of C3H cells, in agreement with the higher expression of PD-L2 observed in these MLCs. Meanwhile, BALB/c MLCs also increased the levels of CD4^+^PD1^+^ and CD8^+^PD1^+^ T cells. Accordingly, adoptive transfer of both C3H and BALB/c MLCs reduced the activation state of T cells (CD62L^−^CD44^hi^), highlighting the involvement of tolerogenic mechanisms. These responses were partially evident in vitro by M2 BALB/c MLCs (CD44 ^hi^CD62L^−^ and CD8^+^PD1^+^ levels) and M2 C3H MLCs (CD8^+^PD1^+^ levels) when co-cultured with MHC-mismatched C57BL/6 T cells (Fig. 5F). However, the ability of M2 MHC-mismatched MLCs to enhance Treg differentiation in vitro was not recorded in vivo, where the involvement of T cell anergy processes appeared to play a greater role as a tolerance mechanism. Although augmentation of Tregs has commonly been associated with improvement in MS models, several current treatments are reported to work through other mechanisms and even to reduce Tregs numbers [57, 58].

Higher expression of TNF-α and IFN-γ in infiltrated T cells was observed after repopulation of the microglia niche with MHC-mismatched MLCs. Unexpectedly, IFN-γ has previously been described to ameliorate EAE by inducing homeostatic adaptation of microglia and attenuation of neuroinflammation, infiltration of inflammatory cells and demyelination [44, 45]. According to our results, the therapeutic role of IFN-γ in EAE was driven by myeloid cells, leading to tolerogenic processes regulated by TGF-β and PD-1 signaling [44, 45]. Conversely, TNF-α, mostly known for its proinflammatory activity, also regulates homeostatic functions in the CNS such as neurogenesis, myelination, BBB permeability and synaptic plasticity [59]. Targeting TNF-α has both beneficial and harmful effects in mouse models of MS, these opposing results being due to the functional dichotomy of its receptors TNFR1 and TNFR2 [59]. Importantly, targeting TNF-α has detrimental effects in MS patients and it was associated with demyelinating events [59–61]. The transient increased expression of TNF-α observed in infiltrating T cells in EAE after treatment with MHC-mismatched MLCs could therefore have both protective and deleterious roles. Accordingly, it is possible that a combined strategy with the selective inhibition of TNFR1 could skew the TNF-α effect towards TNFR2 responses, further reinforcing the therapeutic effect of our approach [59, 62, 63].

Our results show the potential benefit that the allogenic transplant of MLCs could have in the clinic. Both in vitro and in vivo experiments support the ability of BALB/c or C3H M2 MLCs to modulate the immune response of both microglia- and T cell- compartments, promoting tolerogenic mechanisms and leading to a beneficial effect in the autoimmune disease mouse model EAE. While the recovery rates of transplanted MLCs showed quite low percentages, they were enough to unleash the observed therapeutic effects, therefore highlighting the impactful potential this strategy may have in the clinic.

Of note, the structural design of our approach considers several factors to facilitate its possible future translation into clinical practice: (i) microglial depletion compounds are being tested in several clinical trials [64], and even one CSF1R inhibitor has already been approved by the FDA [65]; (ii) since microglia cannot be harvested from humans, our protocol allows obtaining microglia-like cells from peripheral monocytes [12]; (iii) the selection of appropriate healthy donors together with our polarizing protocol confer a suitable anti-inflammatory phenotype to MLCs; (iv) transplanting pre-differentiated cells avoids risks associated with e.g. stem cells becoming tumors [66]; and (v) advancements in the use of the intracisternal administration route would allow the adoptive transfer of MLCs as an ambulatory procedure, admitting repeated infusions if needed without major surgery [67]. Furthermore, rejection of MHC-mismatched MLCs is minimized due to their anti-inflammatory phenotype and their adoptive transfer directly into the CNS, which is considered to have a higher immune tolerance due to its partial isolation from the peripheral immune system [68].

Nonetheless, immune rejection of MHC-mismatched MLCs is a possibility due to the plausible recognition by infiltrated lymphocytes. However, the absence of lymphocytes among transplanted cells (only MLCs) prevents the appearance of pathologies associated with MHC-mismatched transplants such as Graft vs. Host Disease (GVHD) [69]. This kind of pathology could instead be encountered when performing hematopoietic stem cell transplantation, a commonly used repopulation strategy for microglia replacement. Strong immune suppression protocols would be indicated in this case [15]. Conversely, the diverted attention of the immune system towards MHC-mismatched MLCs could contribute itself to the therapeutic effect in autoimmunity. In agreement with our results, other groups have reported an amelioration of autoimmune diseases after a conditioning regimen and peripheral MHC-mismatched mixed chimerism, such as in Type 1 diabetes [70] and EAE [15], although an increase of peripheral Tregs was observed in these settings. Interestingly, a combination of myeloid derived suppressor cells and Tregs prevented acute GVHD severity, highlighting the tolerogenic potential that myeloid cells can hold [71].

Appropriate healthy donors should be selected according to genetic background and predictors for macrophage responses in different inflammatory and disease contexts in a similar way to that previously described [24]. Once identified, peripheral mononuclear cells from either bone marrow or blood samples could be obtained and stored for later differentiation to MLCs and polarization to an M2 phenotype. While the development of cell-therapies encompasses several challenges, the current development of multi-step automated systems for cell therapy manufacturing would facilitate scaling up and reach the pharmaceutical end-user in a cost-effective way [72].

Depending on the disease state, an infusion of MLCs could be sufficient to reset the immune-CNS crosstalk, while other pathologies might need multiple interventions. Further studies would determine the applicability of this strategy in different autoimmune contexts as well as protocol optimizations.

## Conclusions

We propose a microglia repopulation strategy with anti-inflammatory MHC-mismatched MLCs to enhance immune tolerance in autoimmune neuropathologies. While a polarization protocol towards an anti-inflammatory phenotype confers MLCs with beneficial features in a pro-inflammatory disease context, the MHC-mismatch interaction within the host´s CNS promotes additional tolerogenic processes in autoimmunity. Herein, we prove this hypothesis in the MS mouse model EAE, in which we observed an amelioration of disease progression and the promotion of tolerogenic mechanisms in the CNS according to the analysis of both myeloid and lymphoid populations.

## Supplementary Information

Supplementary Material 1

Supplementary Material 2

## Data Availability

The data sets generated for this study are available on request to the corresponding authors.

## Abbreviations

APCs: Antigen-presenting cells
BBB: Blood-brain barrier
BALB/c: BALB/cAnNCtrl
BAMs: BBB associated macrophages
BM: Bone marrow
C3H: C3H/HeNcrl
C57BL/6: C57BL/6NTac
CNS: Central nervous system
EAE: Experimental autoimmune encephalomyelitis
GVHD: Graft vs. Host Disease
i.c: Intracisterna
MLC: Microglia-like cell
MOG: Myelin oligodendrocyte glycoprotein
MS: Multiple sclerosis
OPC: Oligodendrocyte Progenitor Cell
ROS: Reactive oxygen species
Treg: Regulatory T cell
