# Supplementary material for: Allogeneic MHC-mismatched microglia-like cell replacement as a therapeutic approach for multiple sclerosis
Source: J Neuroinflammation. 2026 Jan 8;23:36. doi: 10.1186/s12974-025-03672-4 (PMC12849289; doi:10.1186/s12974-025-03672-4)
Supplement: Supplementary file 1 — Supplementary Material 1 [file 12974_2025_3672_MOESM1_ESM.pdf]

Gene	Forward	Reverse
Arg1	CTCCAAGCCAAAGTCCTTAGAG	AGGAGCTGTCATTAGGGACATC
Cd68	ACCGCCATGTAGTCCAGGTA	ATCCCCACCTGTCTCTCTCA
Gapdh	TGAAGCAGGCATCTGAGGG	CGAAGGTGGAAGAGTGGGAG
Hprt	ACAGCCCCAAAATGGTTAAGG	TCTGGGGACGCAGCAACTGAC
Il10	GGTTGCCAAGCCTTATCGGA	ACCTGCTCCACTGCCTTGCT
IL12a	CTGTGCCTTGGTAGCATCTATG	GCAGAGTCTCGCCATTATGATTC
Il1b	GCAACTGTTCCTGAACTCAACT	ATCTTTTGGGGTCCGTCAACT
IL4	GGTCTCAACCCCCAGCTAGT	GCCGATGATCTCTCTCAAGTGAT
IL4ra	TGACCTCACAGGAACCCAGGC	GAACAGGCAAAACAACGGGAT
Il6	TAGTCCTTCCTACCCCAATTTCC	TTGGTCCTTAGCCACTCCTTC
Mrc1	TGATTACGAGCAGTGGAAGC	GTTCACCGTAAGCCCAATTT
Nos2	GTTCTCAGCCCAACAATACAAGA	GTGGACGGGTCGATGTCAC
Tgfb1	AGCCCGAAGCGGACTACTAT	TTCCACATGTTGCTCCACAC
Tgfb2	TTTAAGAGGGATCTTGGATGGA	AGAATGGTCAGTGGTTCCAGAT
Tgfb3	CGCACAGAGCAGAGAATTGA	GTGACATGGACAGTGGATGC
Tgfbr1	CCTGAAGTTCTAGATGATTCC	CTTCATGGATTCCACCAATAG
Tgfbr2	CCAGGATGAATCTGGAAAAC	TAATCCTTCACTTCTCCCAC
Tnfa	CTGTAGCCCACGTCGTAGC	TTGAGATCCATGCCGTTG

Table S1. Primer sequences used for RT-qPCR

Anti-mouse antibody:	Clone:	Company:	Reference:
I-A/I-E (MHC-II)	M5/114.15.2	BioLegend	107670
CD11b	M1/70	Biolegend	101228
CD11b	M1/70	Biolegend	101216
CD19	ID3	BD Biosciences	562291
CD206	C068C2	Biolegend	141729
CD25	PC61	BD Biosciences	561065
CD25	PC61	Biolegend	102010
CD3	17A2	Biolegend	100216
CD4	RM4-5	Biolegend	100552
CD44	IM7	Biolegend	103026
CD44	IM7	Biolegend	506349
CD45	30-F11	BD Biosciences	552848
CD45.1	A20	Biolegend	110707
CD45.2	104	Biolegend	109832
CD62L	MEL-14	Invitrogen	17-0621-82
CD62L	MEL-14	Invitrogen	45-0621-82
CD8	53-6.7	Invitrogen	48-0081-82
CTLA-4	UC10-4B9	Biolegend	106323
F4/80	BM8	Biolegend	123137
FOXP3	FJK-16s	Invitrogen	25-5773-82
H-2b (H-2Kb, MHC-I)	AF6-88.5.5.3	Invitrogen	17-5958-80
IFN-gamma	XMG1.2	BD Biosciences	554413
IL10	JE55-16E3	BD Biosciences	563277
IL17a	TC11-18H10.1	Biolegend	506904
Ki67	B56	BD Biosciences	567130
LAG3	C9B7W	BD Biosciences	748540
Ly6C	HK1.4	Biolegend	128024
Ly6G	1A8	Biolegend	127614
Ly6G	1A8	Biolegend	560603
MHCII	M5/114.15.2	Invitrogen	46-5321-80
PD-1	29F.1A12	BD Biosciences	568595

Table S2. Antibodies employed for flow cytometry analysis

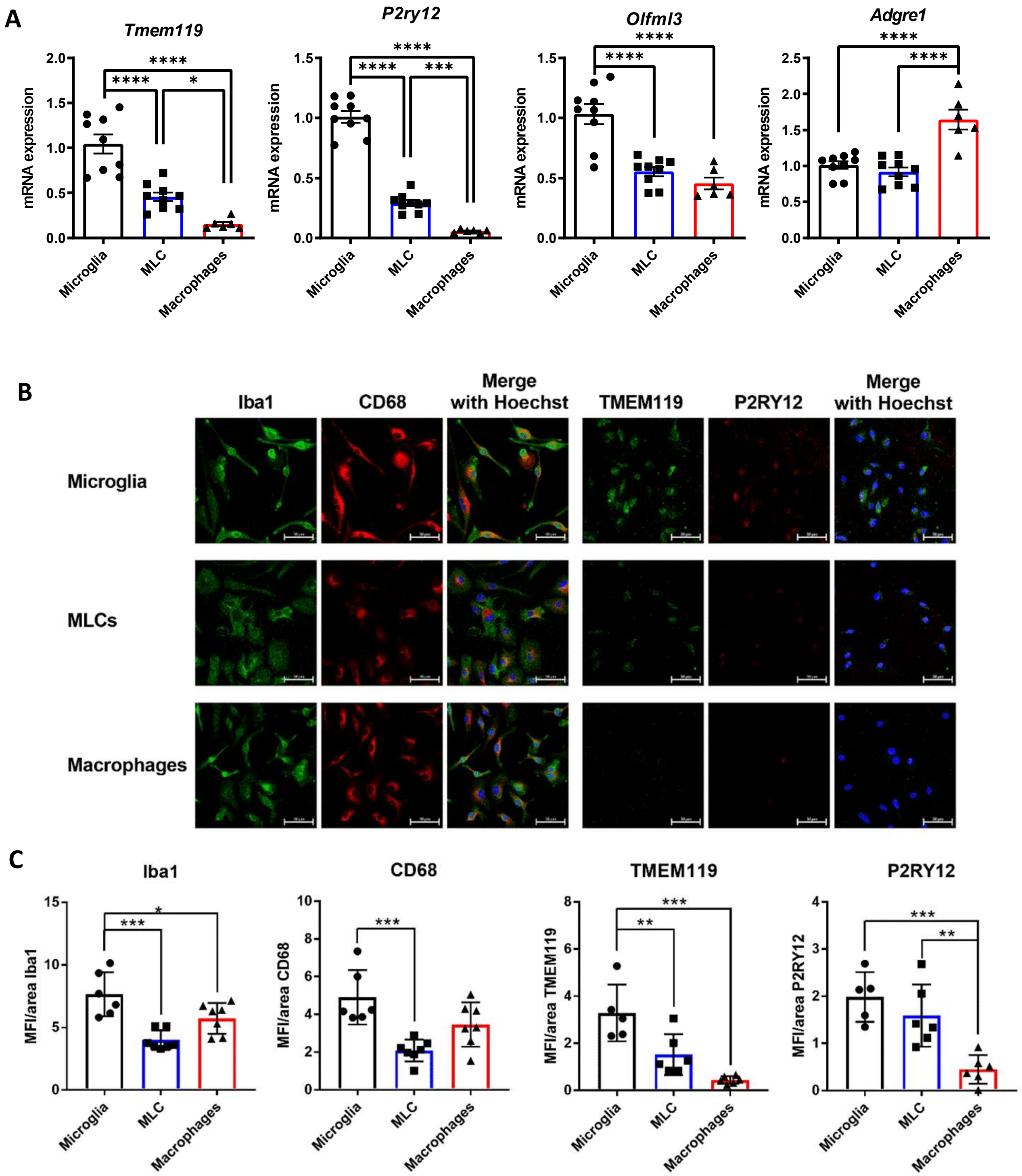

Figure S1. C57BL/6 microglia, MLCs and macrophages were cultured *in vitro* for analysis of myeloid markers. **A)** mRNA expression levels of *Tmem119, P2ry12, Olfml3* and *Adgre1* (Microglia n = 9; MLC n = 9; Macrophages n = 6). **B-C)** Microglia, MLCs and macrophages were cultured and stained with either antibodies against Iba-1 and CD68 (B: left panel) or TMEM119 and P2RY12 (B: right panel) for image acquisition by confocal microscopy (B) and quantification of MFI as indicator of protein expression levels (C). n = 6. Nuclei were stained with Hoechst (blue). Scale bar: 50 µm. Graphs represent data distribution as dots and bars indicating mean ± SEM.

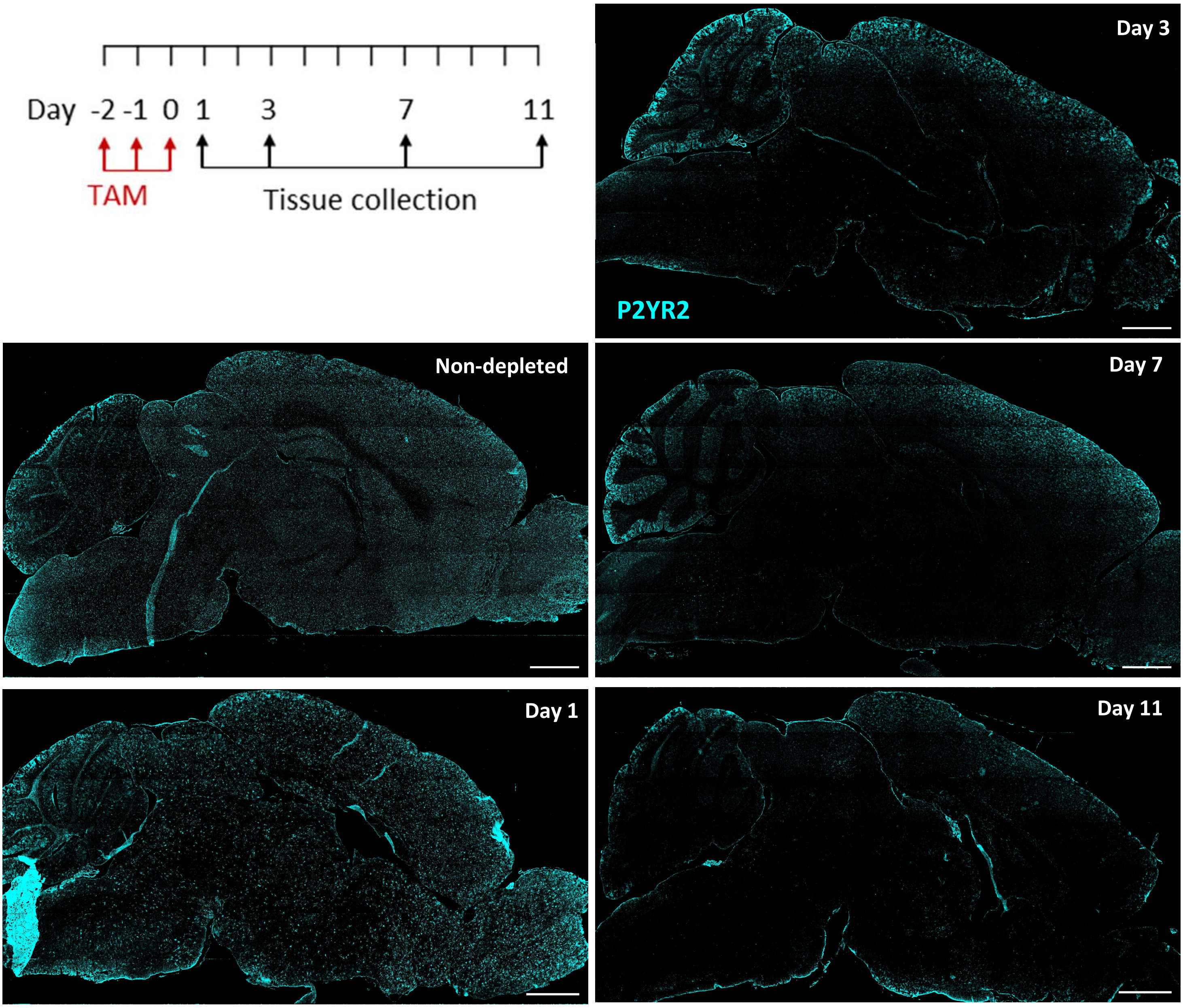

Figure S2. Microglia depletion in $Cx3cr1^{CreER}R26^{DTA}$ mice after tamoxifen treatment for 3 days. Representative images of endogenous microglia (P2YR2) in brain sections by fluorescence microscopy at different timepoints. Scale bar = 1 mm

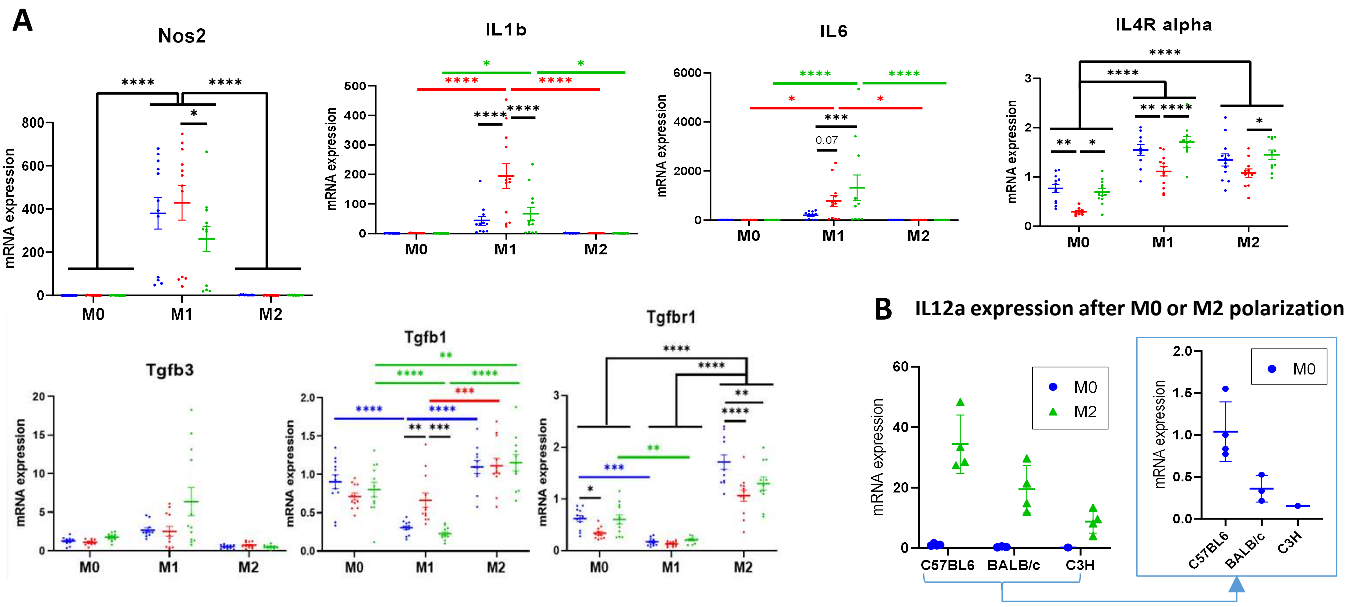

Figure S3. C57BL6 (blue), BALB/c (red) and C3H/He (green) MLCs untreated (M0) or treated for 24 h with LPS/IFNγ (M1) or Il-4, IL-10 and TGF-β (M2). **A)** mRNA expression levels. **B)** mRNA expression levels of IL12a of M0 (blue) or M2 (green) MLCs. Graphs represent data distribution as dots and bars indicating mean ± SEM.

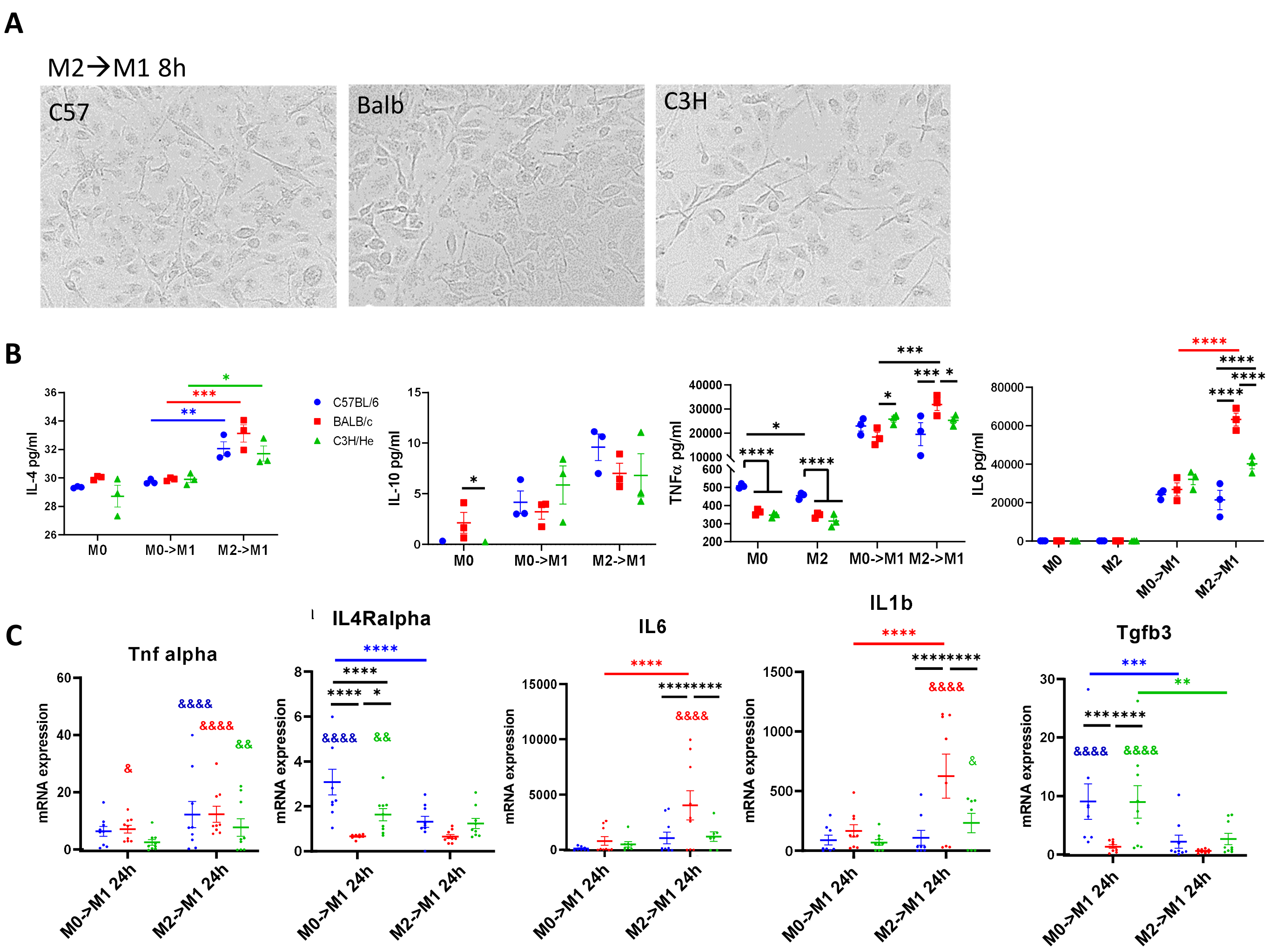

D

vs C57BL6	M0		M1 (↑Nos2, ↑TNFα, ↑IL1β, ↑IL6, ↓Tgfβr2)		M2 (↑Tgfβr1)		M0→M1 (↑ILRα, ↑Tgfβ3, ↓Tgfβr2)		M2→M1 (↑Tgfβ2, ↓Tgfβr2)	
BALB/c	↑IL4 ↓IL4Rα ↓TNFα	↓Tgfβr1 ↑Tgfβ2	↑TNFα ↑IL1β ↑IL6 ↑IL10 ↓IL4Rα	↑Tgfβr2 ↑Tgfβ1 ↑Tgfβ2	↑IL4 ↓IL4Rα	↓Tgfβr1	↑IL4 ↓↓IL4Rα	↑Tgfβr2 ↑Tgfβ2 ↓Tgfβ3	↑IL1β ↑IL6 ↑IL4	↑Tgfβr2 ↑↑Tgfβ2
C3H/He	↓TNFα	↑↑ Tgfβ2	↓TNFα ↑IL6 ↑IL10	↑Tgfβ2	↑IL4		↑IL4 ↓IL4Rα			↑Tgfβ2

Figure S4. MLCs differentiated to M2 phenotype partially retain the anti-inflammatory phenotype when next challenged with a proinflammatory stimulous. C57BL6 (blue), BALB/c (red) and C3H/He (green) MLCs untreated (M0) or treated for 24 h with LPS/IFNγ (M1) or Il-4/IL-10/TGF-b (M2). M0 and M2 MLCs were subsequently treated with LPS/IFNγ (M0→M1, M2→M1). **A)** Representative image of MLCs stimulated towards a M2 phenotype followed by 8h of LPS/IFNγ secondary stimulus. **B)** Secretion levels of IL-4, IL-10, TNF-a and IL-6 by MLCs after 24h of secondary stimulation with LPS/IFNγ, measured by CBA-flow cytometry. **C)** mRNA expression levels after a secondary proinflammatory stimulus. & ($p \leq 0.05$) represents significance in comparison with M0 or M2 treatments without secondary LPS/IFNγ challenge (Two way ANOVA, Tukey´s multiple comparisons test). Graphs represent data distribution as dots and bars indicating mean ± SEM. **D)** Summary table of the variation in the mRNA expression levels of anti- and pro-inflammatory cytokines when comparing BALB/c or C3H/He MLCs with C57BL6 MLCs. ↑ indicates a higher expression compared to C57BL6, and ↓ a lower expression.

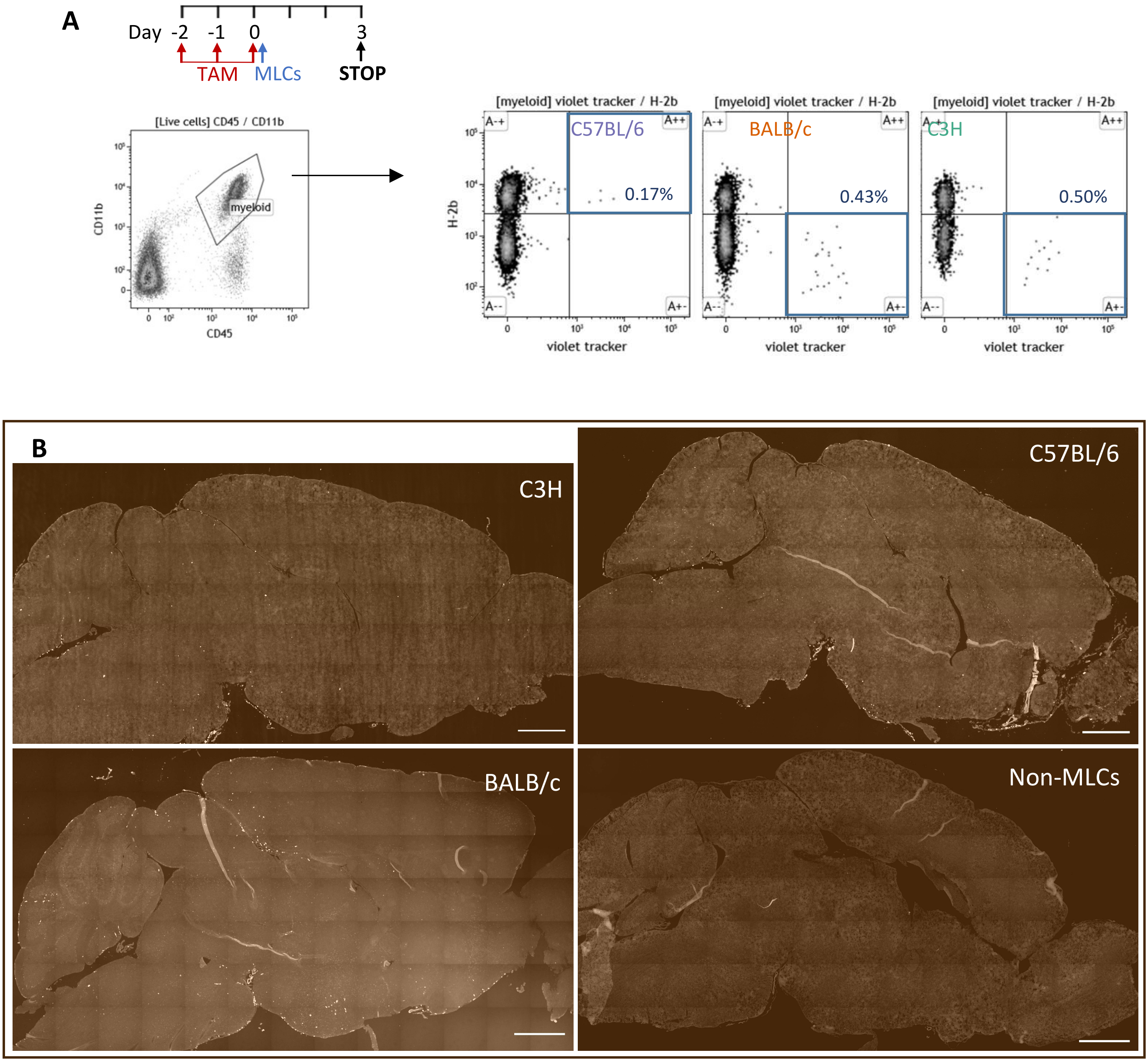

Figure S5. Cx3cr1^{CreER}R26^{DTA} mice (C57BL/6 background) were treated with tamoxifen for 3 days and 5 10^5 M2 C57BL/6, BALB/c or C3H MLCs labelled with violet tracer were administered via intracisterna (ic) the last day of injection. 3 days later, MLCs from the 3 strains where detected in the brain by both flow cytometry (A) and fluorescence microscopy (B). **B)** Representative image of a brain section showing the integration of C57BL/6, BALB/c or C3H MLCs (bright dots) into the brain. Microglia depleted brain without MLC-injection is shown as negative control for violet signal (Non-MLCs). Scale bar = 1 mm

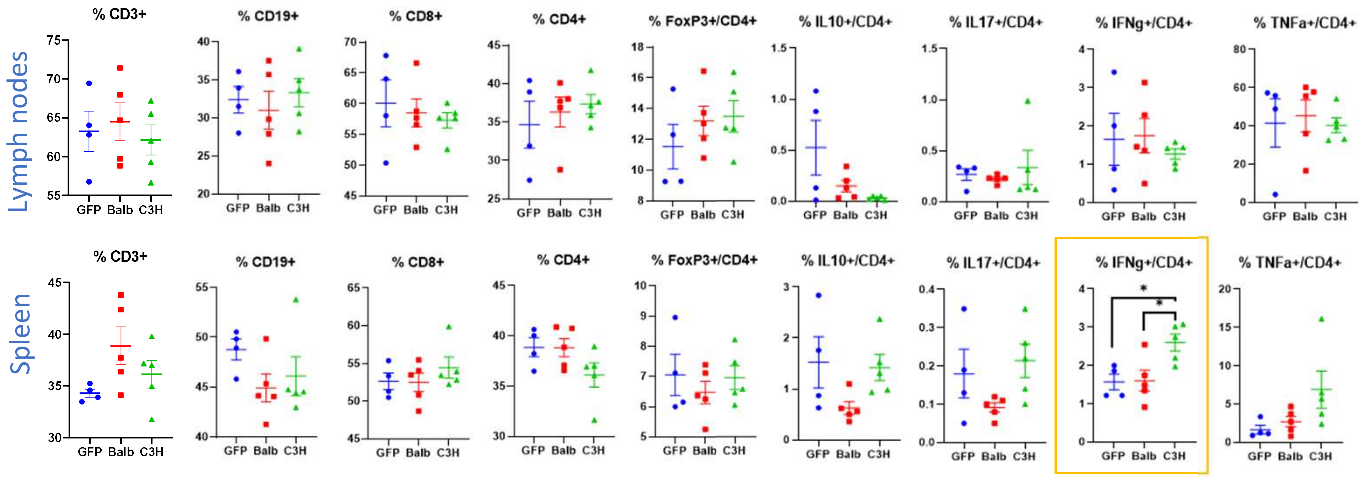

Figure S6. Mice with a C57BL/6 background received food containing PLX3397 for 21 days and, afterwards, M2 GFP-C57BL/6, BALB/c or C3H MLCs were administered ic 3 times each 48h. 10 days later, lymph nodes and spleens were analyzed by flow cytometry. Graphs represent data distribution as dots and bars indicating mean ± SEM. (Spleen % $IFNg^{+}/CD4^{+}$ graph (yellow box) corresponds to data already shown in Figure 4F).

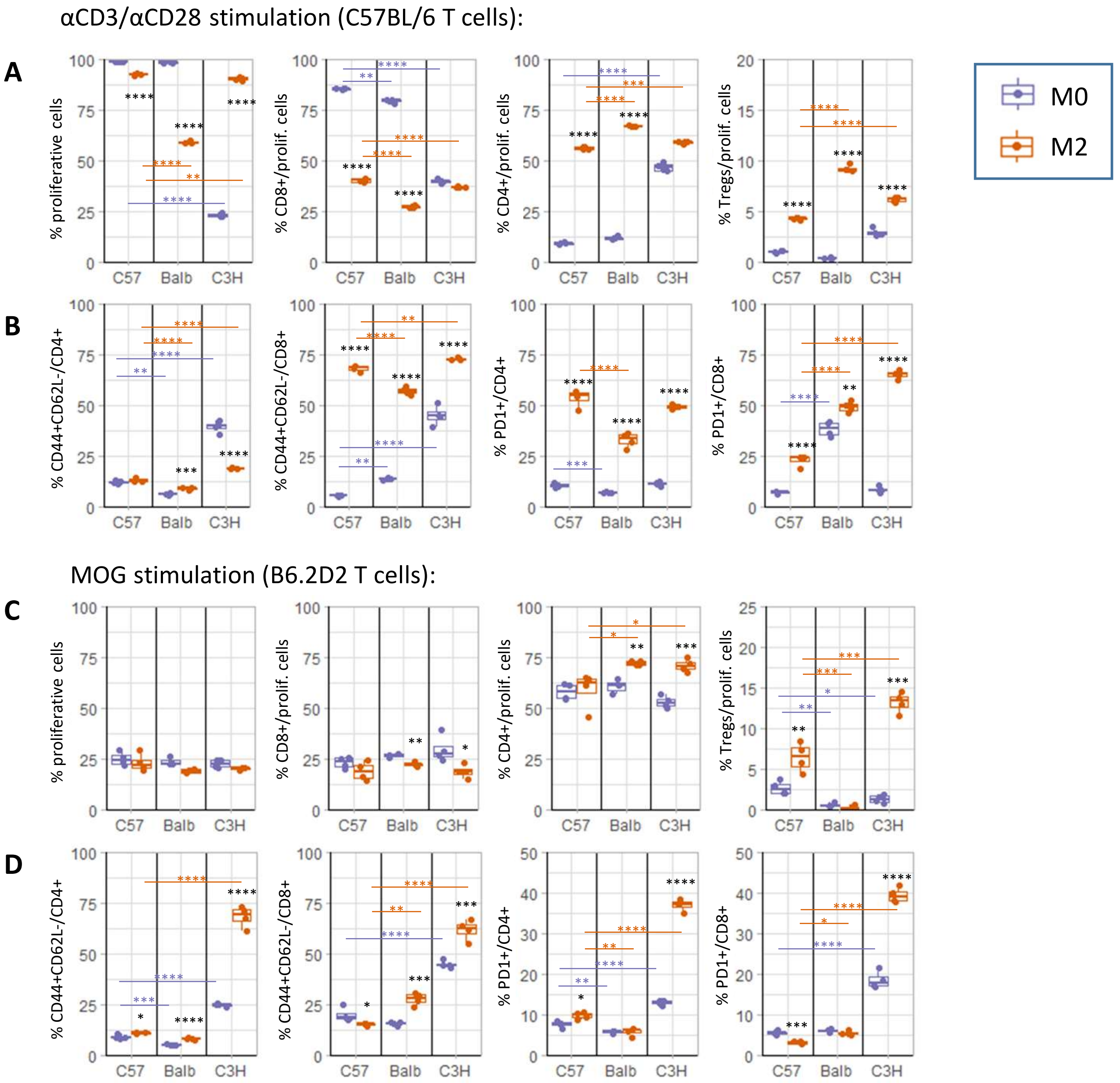

Figure S7. Alternative representation of data from figure 4E-H according to population-specific proliferation dynamics. **A-B)** C57BL6, BALB/c and C3H/He MLCs untreated (M0, blue) or treated for 24 h with Il-4/IL-10/TGF-β (M2, orange) were subsequently cultured with CFSE-labelled T cells collected from C57 mice and stimulated with α-CD3/α-CD28 for 72h for subsequent analysis of T cell proliferation by flow cytometry (n=4). **C-D)** MLCs were co-cultured with T cells collected from B6.2D2 mice and stimulated with MOG(35-55) peptide for 72h for subsequent analysis of T cell population by flow cytometry (n=4). ANOVA, Tukey's multiple comparison test. Data distribution (dots) is summarized as boxplots. Graphs represent a representative experiment of 3 (A,C) or 2 (B,D) independent experiments.

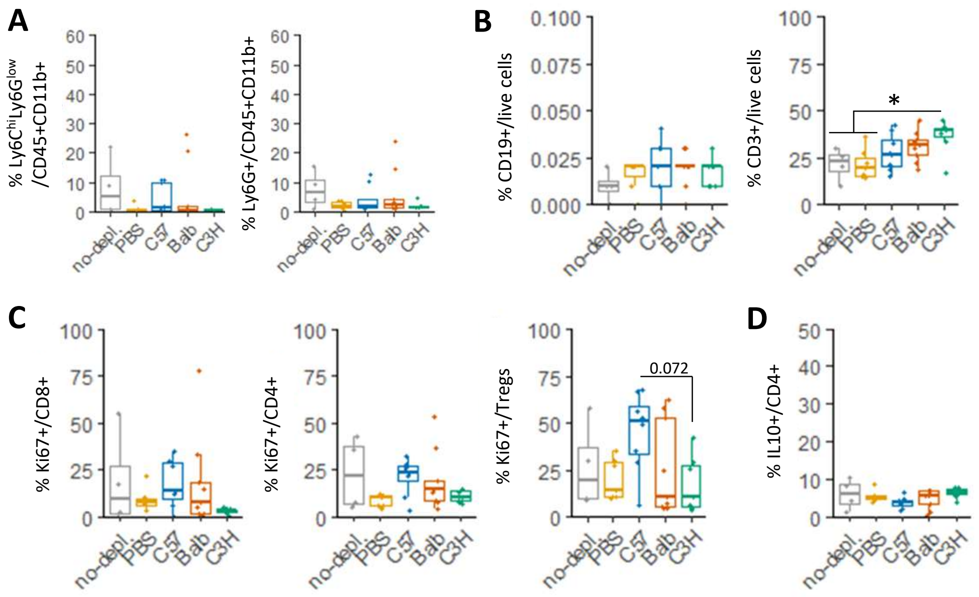

Figure S8. (Related to experiment in Figure 6) Long-term analysis at day 34 of microglia depletion and MLC injection in EAE mice. Flow cytometry analysis of the spinal cords at the endpoint of the EAE. **A)** % of monocytes ($Ly6C^{hi}Ly6G^{low}$) and neutrophils (Ly6G+) among CD11b+CD45+ cells. **B)** Infiltration into the spinal cord of B cells (CD19+) and T cells (CD3+). **C)** Ki67 expression levels of infiltrated T cells. **D)** IL-10 expression levels of infiltrated $CD4^+$ and $CD8^+$ cells. One-Way ANOVA. Data distribution (dots) summarized as boxplots.

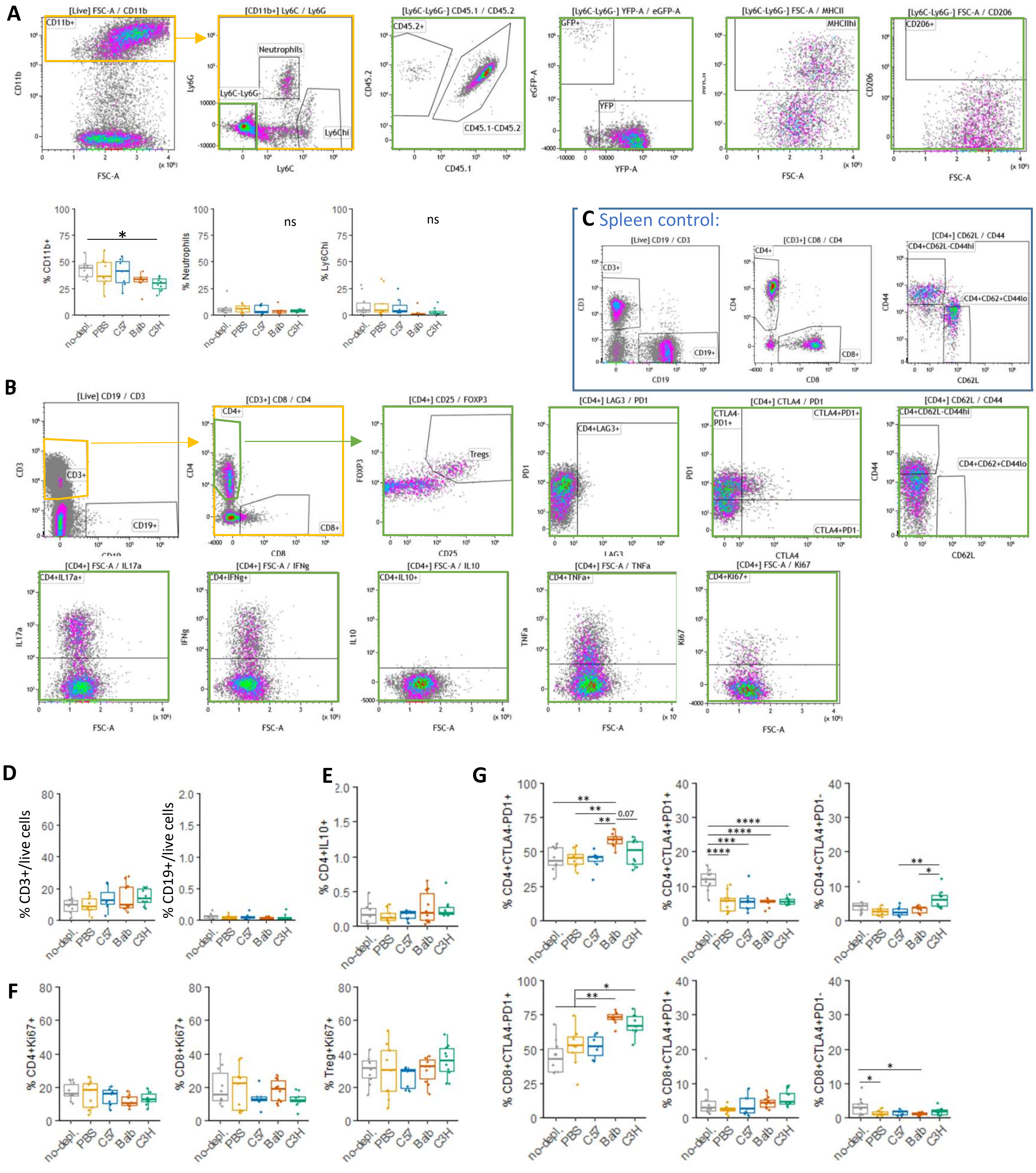

Figure S9. (related to experiment in Figures 7 and 8) 4-months-old CD45.1/CD45.2 Cx3cr1^{CreER}R26^{DTA} and C57BL/6 mice were treated with tamoxifen for 3 days at the onset of EAE symptoms. Microglia-depleted Cx3cr1^{CreER}R26^{DTA} mice received a total number of 10^6 M2 MLCs (or PBS) administered in 3 ic injections: PBS (yellow), GFP-C57BL/6 (blue), BALB/c (orange) or C3H (green). C57BL/6 mice received PBS as negative control of microglia depletion (gray). Flow cytometry analysis of spinal cords at the endpoint of the EAE. **A)** Myeloid cell composition, gating strategy. One-way ANOVA. **B-G)** Analysis of infiltrated lymphocytes. **B-C)** Gating strategy based on isolated cells from spinal cord **(B)** and spleens **(C)**. **D)** % T and B cells. **E)** % CD4+ cells expressing IL10. **F)** % proliferative cells according to Ki67 expression. **G)** % T cells expressing PD1 and/or CTLA4 receptors. Data distribution (dots) summarized as boxplots.

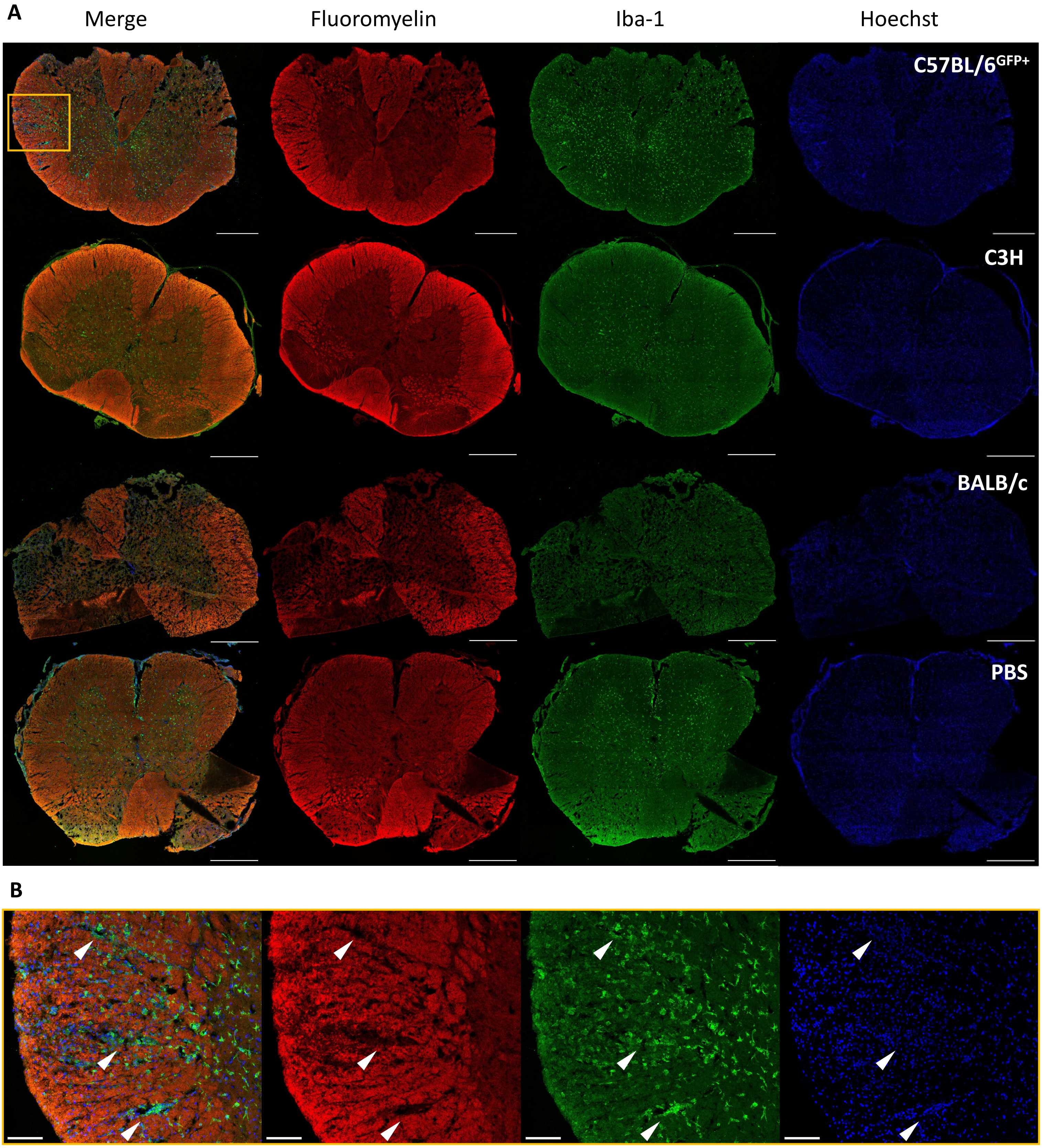

Figure S10. (related to experiment in Figures 7 and 8) 4-months-old CD45.1/CD45.2 Cx3cr1^{CreER}R26^{DTA} mice were treated with tamoxifen for 3 days at the onset of EAE symptoms. Microglia-depleted Cx3cr1^{CreER}R26^{DTA} mice received a total number of 10^6 M2 MLCs (or PBS) administered in 3 ic injections. **A)** Spinal cord sections were labelled with Fluoromyelin (red), Iba-1 (green) and Hoechst (Blue). Scale bar = 500 µm. **B)** Magnification of spinal cord lesions (white arrows) in C56BL/6^{GFP+} recipients. Scale bar = 100 µm.
